# AID-RT: Standardising Artificial Intelligence Documentation in RadioTherapy with a domain-specific model card^☆^

**DOI:** 10.1016/j.phro.2026.100940

**Published:** 2026-03-06

**Authors:** Ana M. Barragán-Montero, Margerie Huet-Dastarac, Silvia M. Herranz-Hernández, Benjamin Tengler, Emma Skarsø Buhl, Arthur Galapon, Carlos E. Cárdenas, Marco Fusella, Geoffroy Herbin, Yvonne de Hond, Franziska Knuth, Ciaran Malone, Peter van Ooijen, Charlotte Robert, Michele Zeverino, Coen Hurkmans, Tomas Janssen, Stine Sofia Korreman, Charlotte L. Brouwer

**Affiliations:** a UCLouvain – Institut de Recherche Expérimentale et Clinique, Molecular Imaging Radiotherapy and Oncology (MIRO), Brussels, Belgium; b University of Tübingen, Department of Radiation Oncology, Section for Biomedical Physics, Tübingen, Germany; c Danish Center for Particle Therapy, Department of Clinical Medicine, Aarhus University, Aarhus, Denmark; d University of Groningen, University Medical Center Groningen, Department of Radiation Oncology, Groningen, the Netherlands; e University of Alabama at Birmingham, Department of Radiation Oncology, Birmingham, AL, USA; f Radiation Oncology Department, Policlinico Abano Terme, Abano Terme, Italy; g Ion Beam Applications, Louvain-la-Neuve, Belgium; h Catharina Hospital Eindhoven - Department of Radiation Oncology, Eindhoven, the Netherlands; i Erasmus MC Cancer Institute, University Medical Center Rotterdam, Rotterdam, the Netherlands; j Department of Medical Physics, St.Luke’s Radiation Oncology Network, Dublin, Ireland; k Department of Radiation Oncology, Gustave Roussy, Villejuif, France; l Institute of Radiation Physics - Lausanne University Hospital, Lausanne, Switzerland; m Department of Electrical Engineering and Department of Applied Physics and Science Education, TU Eindhoven, Eindhoven, the Netherlands; n Department of Radiation Oncology, The Netherlands Cancer Institute, Amsterdam, the Netherlands

**Keywords:** Artificial intelligence, Model card, Standardisation, Documentation

## Abstract

**Background and Purpose:**

Insufficient documentation of artificial intelligence (AI) models remains a widespread issue, which hampers reproducibility in research environments and safe integration in clinical departments. Our goal was to develop a standardised, structured, and domain-specific reporting framework tailored to AI models in radiotherapy (RT), enhancing transparency and accountability.

**Methods:**

A working group was formed after the ESTRO Physics Workshop 2023, *“AI for the Fully Automated Radiotherapy Treatment Chain”,* comprising 16 experts from 13 institutions*.* We reviewed existing initiatives for AI model and data reporting and drafted an initial template, which was sent for review to all participants. Three popular RT applications were selected to define task-specific fields: synthetic CT, segmentation, and dose prediction. Five review rounds were performed, where suggested changes were voted in a shared online document. Unclear fields and conflicting votes were discussed at online meetings, and consensus was reached by majority voting.

**Results:**

The final template included 6 sections: 0) Card metadata, 1) Model basic information; 2) Model technical specifications (i.e. architecture, software and hardware); 3) Training data, methodology, and information; 4) Evaluation data, methodology, and results (a.k.a commissioning for clinical models); and 5) Other considerations, including ethical use, risk analysis, and monitoring. It is publicly available as a downloadable document template and as an interactive web-based form to facilitate information entry.

**Conclusions:**

We proposed a practical, consensus-driven template tailored to the unique requirements of AI models in RT, with applicability in both research and clinical environments, conveying the key information required for informed use.

## Highlights

• Consensus-based model card for documenting artificial intelligence in radiotherapy.
• Initiative from ESTRO Physics Workshop 2023.
• Covers model and data aspects for training, evaluation, and ethical use.
• Enhances transparency and safe clinical integration of artificial intelligence.
• Publicly available template for research, clinical, and educational use.

## Graphical abstract

**Figure:**
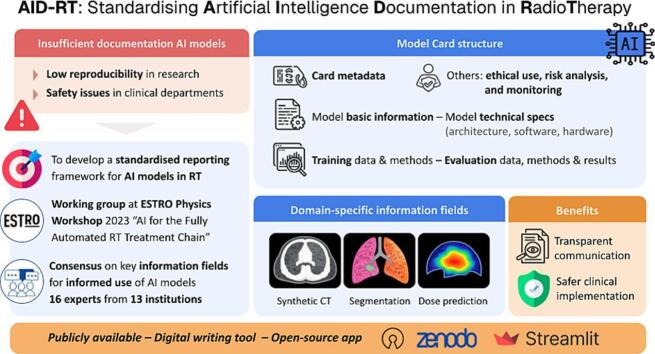

## 1 Introduction

Artificial intelligence (AI) is rapidly transforming the field of radiotherapy (RT), with a growing number of research studies that explore AI for RT tasks and with models increasingly embedded into clinical workflows [1], [2], [3], [4]. As these models transition from development to deployment, the need for clear, accessible reporting, and transparency of models become critical, both in research and clinical environments. In research, transparency is essential to ensure reproducibility of results, meaningful benchmarking, and to favour independent validations by external groups. In clinical settings, it is important to understand and compare the scope, requirements and limitations of different models in order to assess their reliability and applicability to patient care.

Particularly for clinical-grade AI models, regulatory bodies have begun taking steps to enhance transparency and safety. Broad frameworks such as the EU AI Act [5] and more healthcare-specific instruments such as the EU Medical Device Regulation (MDR) [6], UK MHRA guidelines [7], or the recent draft guidance for AI-enabled device software from FDA [8], emphasise transparency and monitoring throughout the model lifecycle. This includes requirements for documentation related to training methodology, performance reporting, risk management, intended use, or model updates. However, as yet, these frameworks lack clear instructions and standards for end users on how this documentation should be structured and reported in practice.

Efforts to standardise AI documentation have emerged across disciplines, including general-purpose model and/or data reporting tools (e.g., model cards [9] and datasheets [10]) and healthcare-specific guidelines. However, most are either too generic to fully capture domain-specific needs or too focused on research reporting to be practical in clinical settings. Most importantly, while several initiatives focus on general healthcare, medical imaging or radiology [11], [12], there is currently no standard approach that is specific to RT [13]. As a result, each team often re-develops documentation procedures from scratch, which is an important limitation for clinical deployment, leading to inefficient and resource-intensive implementation processes. Thus, inconsistent and insufficient documentation remains a widespread issue, undermining trust, reproducibility, and safe clinical integration, while also limiting AI literacy and adoption among clinical teams [14], [15], [16], [17].

In this context, there is a pressing need for a structured, domain-specific reporting format tailored to radiotherapy AI models, analogous to the role that DICOM (Digital Imaging and Communications in Medicine) [18], [19] has played in standardising medical image data and improving interoperability. This was highlighted as one of the key priorities for our community at the ESTRO Physics Workshop 2023, *“AI for the Fully Automated Radiotherapy Treatment Chain”.* In response, a working group on behalf of the ESTRO AI Focus group, comprising the authors of this paper, was established to develop a standardised reporting framework aimed at addressing the documentation needs of both research and clinical RT communities.

This manuscript reviewed the existing initiatives for AI model and data reporting, discussed their potential and limitations, and introduced a new, domain-specific AI model card for radiotherapy: a practical, consensus-driven template tailored to the unique requirements of AI models in this field, conveying the key information required for informed use. The latter is a key factor ensuring effective human oversight, in line with the newly established EU AI Act regulation. The consensus was developed through collaboration among researchers, clinicians, and vendors to ensure broad relevance and applicability. Thus, the presented model card is intended to support multiple stakeholders by: 1) facilitating reproducibility for researchers developing and benchmarking AI models, 2) enabling easier model comparison and safer deployment for clinical teams, and 3) supporting clear communication of model information to end users and building trust for vendors. The model card template is publicly available as a Microsoft Word document on Zenodo (zenodo.org/records/15336016) and as an interactive digital template to facilitate information entry at rt-modelcard.streamlit.app. In addition, we provided, in Supplementary Material, illustrative examples of our model card for three of the most common AI applications in the field: synthetic image generation, segmentation (also referred to as contouring), and dose prediction.

## 2 Methods

### 2.1 State-of-the-art of AI documentation − potential and limitations

In the last few years, several initiatives have emerged across disciplines to address the broader challenges of AI model transparency through comprehensive documentation. We distinguished here between general-purpose tools (Table 1) and healthcare specific guidelines (Table 2). Supplementary Table S1 compares AID-RT features with respect to existing AI documentation frameworks.

**Table 1:** Summary of some of the most popular generic purpose guidelines, templates, and tools for model and/or data reporting. More of them can be found in reference [21].

Tool	Reference	Summary
ML Canvas	Dorard, 2015, https://www.ownml.co/ machine-learning-canvas [43]	Generic and simple template for a model card, available in different formats: PDF, Word, html, OpenDoc.
Model Card	Mitchell et al. 2018 [9]	First model card template, by Google. Generic to any AI model. Includes model details, train / evaluation data, and performance.
Data statements for NLP	Bender et al. 2018 [44]	Schema of information to include in long and short form data statements to address bias and ethical issues in natural language processing (NLP).
Factsheets	Arnold et al., 2019 [20]	Collection of relevant information (facts) to promote transparency during the creation and deployment of an AI model, by IBM.
Datasheets	Gebru et al. 2018 [10]	First standard for datasets, by Microsoft. Generic to any dataset. Includes motivation, composition, collection process, cleaning, labelling, uses, maintenance, etc.
Data cards	Pushkarna et al. 2022, [45]	Structured summaries of essential facts for ML datasets needed by stakeholders across a dataset’s lifecycle for responsible AI development in industry and research.
Dataset nutrition label	Holland et al., 2018; Chmielinski et al. 2022; https://datanutrition.org/[46], [47]	Framework for standardized data analysis including an overview of dataset “ingredients” before AI model development. Applicable to different domains and data types.
AI ethics label	Hallensleben et al. 2020, [48]	Similar approach to the ones currently used in food and energy-efficient labelling. It communicates high-level information through simple color scales and ratings (e.g. A-F).
HuggingFace model creator	Ozoani 2022, hugginface.co/docs/hub/en/model-card-annotated	Tool to help operationalize model cards and create your own for specific applications.

**Table 2:** Summary of health-care specific reporting guidelines and checklists.

Tool	Reference	Summary
CLAIM	Mongan et al. 2020 [12]	Checklist for transparency in research papers involving AI in medical imaging.
MI-CLAIM	Norgeot et al. 2020 [49]	Checklist with minimum information about clinical artificial intelligence modeling.
Model Fact Labels	Sendak et al. 2020 [50]	1-page with relevant information to support clinicians for AI-based decision making.
MINIMAR	Hernandez-Boussard et al. 2020 [51]	Checklist for MINimum Information for Medical AI Reporting.
SPIRIT-AI CONSORT-AI	Cruz Rivera et al. 2020 [52], [53]	Guidelines for clinical trials protocols and reports for interventions involving AI.
CLAMP	Naqa et al. 2021 [54]	Methodology in sufficient detail to allow replication in publications.
STARD-AI	Sounderajah et al. 2021 [55]	AI version of the Standards for Reporting of Diagnostic Accuracy Study checklist.
DECIDE-AI	Vasey et al. 2022 [56]	Reporting Checklist for decision support systems (academic).
PRISMA-AI	Cacciaman et al. 2023 [57]	Guidelines for systematic reviews and *meta*-analysis of AI interventions.
CLEAR	Kocak et al. 2023 [28]	CheckList for EvaluAtion of Radiomics research (CLEAR).
TRIPOD + AI	Collins et al. 2024 [11]	Reporting of studies that develop a prediction model or evaluate its performance.
PROBAST + AI	Moons et al., 2025 [27]	Quality, risk of bias, and applicability assessment for prediction models using AI.
FUTURE-AI	Lekadir et al. 2025 [29]	Guideline for trustworthy AI tools in healthcare. Broad content covering design, development, regulation, deployment, and monitoring.
CHAI ARC	Coalition for Health AI 2024 [58]	Assurance Reporting Checklist (ARC) for Trustworthy Health AI.
AAPM / ESTRO	Hurkmans et al. 2024 [31]	Different statements and recommendations, split by application-specific in RT.

#### 2.1.1 General-purpose tools for model and data reporting

General-purpose tools such as Model Cards [9], Datasheets [10], and Factsheets [20] offer templates for describing model and/or data characteristics and intended use. Table 1 provides a summary of the most well-known tools within this category, although more of them can be found in the HugginFace Guidebook [21]. These templates typically walk the user through key aspects of model development and deployment, such as intended use and users, model architecture, training data, performance metrics, ethical considerations, and known limitations. They are generally structured in a modular or question-based format (e.g. For what purpose was the dataset created? – in Datasheets [10]). Most of these tools are designed to be concise, typically around one page, and aim to convey the most critical information to support informed use. While these tools are valuable starting points, their generic nature often leaves too many formatting and content decisions to the user, making them not suitable in terms of interoperability, product comparison and safe clinical implementation in highly specialised domains such as RT. Recently, De Biase et al. [22] performed a study applying three such frameworks, ML Canvas [23], Model Cards [9], and Datasheets [10], to a specific AI model for a typical RT problem (i.e. automatic segmentation of tumor volume from multimodal PET/CT images for oropharyngeal cancer patients). They concluded that while these tools can be useful, they still require fine-tuning to meet the specific needs of the field.

Indeed, the complexity of medical data and the level of accuracy required for clinical-grade AI models are rather high. This calls for detailed technical documentation and standardisation to support interoperability and meaningful comparison. For example, in AI models involving medical imaging modalities such as CT, MRI, or PET, factors such as scan acquisition parameters, field-of-view, patient positioning, and reconstruction settings, can significantly impact model performance and generalisability [24], [25], [26]. These imaging-specific variables are often overlooked in generic reporting templates, yet they are critical for clinicians and developers to assess a model’s applicability to their own local workflows, and to design proper monitoring and quality assurance tools after clinical introduction. Another limitation in some of the existing generic tools is the lack of detailed metadata fields to support proper model lifecycle monitoring. Including specific fields for tracking model versioning and reasons for change is critical for model deployment within clinical settings, where traceability is essential. In addition, defining standardized performance metrics is key to ensuring model and version comparability.

#### 2.1.2 Health-care specific reporting guidelines

In parallel to the previously described generic tools, numerous *healthcare-specific reporting guidelines* have emerged in recent years. These are often presented as checklists designed to improve the transparency and completeness of AI reporting in journal articles and clinical trials. Examples include CLAIM [12], TRIPOD + AI [11] or PROBAST + AI [27], among many others. Table 2 provides a summary of these initiatives. Such guidelines play an important role in promoting transparency in research environments, but are not intended to support model deployment or routine use in clinical settings.

Compared to general-purpose tools, these checklists are often better aligned with medical use cases and may include some domain-specific elements. For instance, CLAIM is tailored to medical imaging and includes a dedicated item for the “Image acquisition protocol,” while CLEAR [28], developed for radiomics, includes specific fields relevant to that domain. However, these tools also present important limitations in terms of standardisation, clarity, and practical usability. A recent study [13] evaluated the inter-rater reliability of the TRIPOD and PROBAST guidelines by asking six co-authors to independently score ten research articles in the RT domain, specifically focused on segmentation and radiotherapy planning. The results showed that agreement among reviewers was no better than chance, highlighting significant challenges related to subjective interpretation due to ambiguous phrasing and compound questions. These findings raised concerns about the objectivity and reproducibility of such checklists when applied to AI research in radiotherapy and related fields.

Besides these research-oriented checklists, there are a few complementary initiatives that are more directly applicable to clinical practice and provide broader recommendations for the clinical development, validation, and implementation of AI tools (Table 2, bottom). Documents such as FUTURE-AI [29], CHAI-ARC [30], and the recent joint AAPM/ESTRO guidelines [31] offer valuable guidance on the full model lifecycle. Yet, despite their relevance to both researchers and clinicians, these frameworks do not propose a structured documentation format that could be adopted routinely across vendors, institutions or projects.

### 2.2 Our approach: model card development and consensus process

The working group was established after the ESTRO Physics workshop in October 2023, comprising 16 experts in the field of AI for radiotherapy from 13 institutions, with different backgrounds including research, clinical medical physics, and industry. The goal of the group was to reach a consensus on the content of a model card tailored to AI models in RT, with applicability in both research and clinical environments. For the latter, the group decided to consider the particularities of both in-house developed models and commercial models. It is important to note that, while the group’s objectives align with current regulatory efforts to enhance transparency through documentation, regulatory compliance itself was outside the scope of this initiative. Regulatory requirements may change with time and differ between countries. The proposed model card was not meant to fulfill or replace any regulatory documentation. Instead, the aim was to create a long-lasting and complementary template with content and structure grounded in expert consensus, specifying key information that should be communicated for any AI model in RT for its informed use.

The first step was to perform a literature review (Section 2.1) on existing tools and guidelines, which was complemented with examples gathered by several participants from internal documentation and commissioning reports of AI models implemented in their institutions. Based on this material, an initial model card was proposed and sent for review to all members of the working group. Three sub-groups were created, each of them analysing the applicability of the model card in one of the three most commonly deployed AI applications in RT: image-to-image translation (synthetic CT generation), segmentation, and dose prediction. These groups could add as many task-specific information fields to the initial draft of the model card as needed.

Five review rounds were performed, where suggested changes by individual participants or sub-groups were voted in a live shared Google doc by all participants. Details of the entire process are presented in Supplementary Table S2. Unclear fields and conflicting votes were discussed at online meetings, and consensus was reached by majority voting. While the first three and last review rounds were done among the initial group, the fourth (pre-final) review round included external reviewers (i.e. not familiar at all with the developed prototype so far) from two clinics and three companies (i.e. Varian Medical Systems from Siemens Healthineers, RaySearch Laboratories, and Therapanacea). Note that the initial working group formed during the ESTRO Physics Workshop consisted primarily of clinical medical physicists and researchers; only one vendor representative was present at that stage. To avoid potential bias due to commercial interests, we deliberately postponed broader vendor involvement until a solid proposal was achieved (fourth review round). We wanted this initial group to define, without any commercial influence, what research and clinical teams would want to ask for in a model card.

## 3 Results

The final version of the model card contained six sections, described below, and a total of 198 information fields. It is publicly available on Zenodo as a Microsoft Word file (https://zenodo.org/records/15336016) and as a digital template at https://rt-modelcard.streamlit.app/. The Zenodo version has a unique digital object identifier (DOI), enabling us to upload new future versions as the information fields may evolve. The information fields were classified as “REQUIRED”, indicated by an asterisk (*), or “OPTIONAL”. The former amounted to 131 fields (66%), and included fields that are considered crucial for interoperability and safety (e.g. “Model scope*”, “Clearance*”, or “Observed limitations*”); while the latter (67 fields, 33%) referred to those fields that enhance transparency but might not be critical, as well as fields that could be difficult to disclose due to proprietary constraints, particularly in the case of commercial models. Nonetheless, for research and/or open-source models, we strongly encourage completion of all fields to maximise transparency and reproducibility.

Generic fields, i.e. not specific to any of the selected RT tasks, represented the majority of fields in the model card (140 fields, 70%) and had white background in the Word file, while task-specific fields (only 30%) were indicated with a color code: orange for image-to-image conversion, green for segmentation, and blue for dose prediction). Generic fields were initially derived from the Hugging Face template [32], [33], itself inspired from Google’s prototype [9], and were subsequently refined and expanded to accommodate RT-specific requirements and terminology.

In the digital template, the content is automatically adapted for the selected task with the corresponding task-specific fields, or only generic ones in case that “other” is selected. Task-specific fields were mainly present in the sub-sections for technical characteristics of the training and evaluation data, or in the quantitative performance evaluation.

Feedback from vendors included the change of required contact information fields to optional to avoid potential GDPR issues for publicly available model cards, and modifying certain required fields regarding model architecture and training data to protect proprietary information. These proposals were discussed and voted on during the final review round (restricted to the initial group), which resulted in a compromise: contact and some architectural information fields were made optional, while critical details such as the loss function and key specifications of the training data (e.g., scan parameters) remained mandatory.

The six sections of the final model card comprised:

1) ***Card metadata***. This section includes administrative and versioning information about the model card itself, such as the creation date, version number, and a summary of changes between versions. Including a DOI is also encouraged to support persistent referencing and traceability. Including card-specific versioning information enables us to track how model documentation evolves over time (e.g. a new limitation is found when using the model and needs to be added, a new evaluation on an external dataset is added).
2) ***Model basic information***. This section contains the core descriptive and contextual information necessary to uniquely identify, track, and responsibly use the AI model. It includes fields for model identification and traceability (e.g. name, version number, DOI, and creation date). It also covers details related to model development and provenance, such as the institutions and individuals involved, contact information, software license, code/model availability (e.g. GitHub repository), and potential conflicts of interest, which are important for transparency and enable model reuse or collaboration. Finally, it provides key information for informed use and safety, including intended users, model scope, anatomical application, regulatory clearance, and observed or potential limitations. These fields help end-users understand the context, appropriate use, and potential risks of deploying the model, whether in research or clinical environments.
3) ***Technical specifications***. This section provides a technical breakdown of the model, and it is divided in three sub-sections: 2.1. Model overview, 2.2. Learning Architecture, and 2.3. Hardware/Software requirements. It is important to note that the group defined a “model” as the entire sequence of operations that constitute an AI-based application, including pre-processing, the main learning architecture (i.e. the component with trainable parameters), and post-processing. In other words, the model is not limited to the trainable core, but rather includes all steps required to receive inputs, perform inference, and generate (clinically) usable outputs. This definition reflects our view that comprehensive documentation of the entire pipeline is essential for safety, reproducibility, and clinically appropriate use, beyond solely describing the trainable component. Thus, the “Model overview” sub-section outlines the complete pipeline used for inference, including fields such as pipeline summary, input/output specifications, pre- and post-processing. This allows users to understand how data flows through the system and which non-trainable operations may affect model behavior or performance. On the other hand, the “Learning Architecture” sub-section focuses exclusively on the machine learning component(s), including detailed technical specifications, such as the number and type of inputs/outputs, loss function, regularization, uncertainty quantification, and explainability methods. Where models include multiple components (e.g. cascaded or multi-agent systems), each architecture should be described independently as sub-sections in 2.2. Finally, the last sub-section collects the software and hardware requirements to run the model at inference time. It includes details about necessary libraries and dependencies, expected inference time, hardware recommendations, and installation instructions. In addition, fields for estimating the environmental impact of model inference and training (e.g. carbon emissions) are included to support sustainability efforts and responsible AI development [34].
  Note that nowadays, certain AI-based applications are composed of several AI models. For instance, an autocontouring application where data is routed to site-specific AI models for different anatomical locations (e.g brain, pelvis, breast, …). In this case, we encourage that a model card is filled out for each individual model within the application.

4) ***Training data, methodology, and information***. This section provides detailed information about how the model was trained, including the dataset specifications and validation strategy. For fine-tuned models, it begins with metadata about the base model, including its associated model card and tuning technique.
  The “Training Dataset” portion is subdivided into three parts to help organize the large volume of information. The *General information* part includes details such as dataset size, sources, acquisition period, inclusion/exclusion criteria, and data augmentation strategy. *Technical characteristics* describe image resolution, patient positioning, acquisition protocol, scan parameters, and task-specific variables for dose prediction such as beam configuration or target volume prescriptions. An important required field here is the Reference standard and QA, which refer to the method used to obtain reference ground truth to train the model (e.g. delineation guidelines, planning protocols, etc.) and its QA (e.g. how inter-observer variability for a given contouring guideline in a segmentation task was handled). The last sub-section, *Patient demographics and clinical characteristics*, includes variables such as age, sex, TNM staging, ICD codes, and anatomical target volumes. Wherever relevant, we encourage reporting median and range values to summarize training data characteristics.

The section also includes a description of the validation strategy, including data partitioning and model selection criteria, along with (optional) training hyperparameters such as weight initialization, number of epochs, optimizer, learning rate, and inference strategy. If available, training and validation loss curves can be included to help assess training stability and model generalization.

5) ***Evaluation data, methodology, and results / commissioning*** aims to document how the model was evaluated. To allow evaluations on different datasets, this section can be repeated multiple times with specific indexes. Evaluation refers to general reporting of model performance; it can be done using public or private datasets, but not tied to a specific clinical workflow. Commissioning refers to model evaluation performed within a specific clinical environment using clinic-specific data, with the aim of supporting local clinical use. However, the same structure is applied in both cases. Each evaluation instance starts by including metadata about the evaluation dataset, using the same structure as the training dataset (e.g. size, image acquisition details, patient characteristics, etc.). Then, the section is split into *Quantitative and Qualitative evaluation*, with further subsections adapted to the task being evaluated. Here is mainly where the task-specific fields play an important role. For quantitative evaluation, multiple metrics are proposed depending on the task, such as image similarity metrics (e.g. MAE, SSIM) for synthetic CT generation, geometric metrics (e.g. Dice score, Hausdorff distance, etc.) for segmentation, and dosimetric metrics (e.g. gamma passing rate, DVH-based, etc.) for dose prediction. For each quantitative metric reported, it is mandatory to include the mean, range and standard deviation, together with a description of the computation of the metric and the volume or region it applies to, and the image registration method (if any). Providing per-sample results and visual summaries (e.g. boxplots, histograms) is optional but highly recommended and can be included as an appendix. The final choice of the metrics to be reported is left to the user, but we highly recommend using at least two of the proposed ones. For qualitative evaluation, human-centered metrics such as Likert scoring, Turing test, time savings, or explainability insights may be reported, together with the evaluators' information (e.g. number, background, etc.).
6) ***Other considerations.*** This section addresses optional additional information about broader ethical and operational aspects. It includes any relevant responsible use and ethical considerations, such as concerns around data privacy, fairness, and potential societal impacts of the model’s use. These considerations should go beyond, and not repeat, content already disclosed under “Observed limitations” and “Potential biases” in Section 1. Additionally, a link to a risk analysis may be provided, ideally in the form of a Failure Mode and Effects Analysis (FMEA) [35], [36] or an equivalent framework. Finally, this section may include recommendations for post-market surveillance or live monitoring, such as procedures for tracking performance, handling feedback, and updating the model if needed.

## 4 Discussion

This work reviewed the potential and limitations of the existing tools and guidelines for AI model and data reporting, and proposed a domain-specific AI model card for radiotherapy: a structured, consensus-driven template aimed at comprehensively documenting AI models across both research and clinical settings.

While the current template contained specific fields from only three tasks (i.e. synthetic CT, segmentation, and dose prediction), it could be extended to other tasks within radiotherapy or even to related medical domains, such as radiology. A valuable and immediate extension would target RT tasks where AI is playing an important role, such as image reconstruction, quality assurance, or outcome prediction. However, such extensions must be approached with caution to avoid the pitfalls of overly generic tools, as discussed in Section 2.1. Our consensus view is that model cards should not follow a “one-size-fits-all” approach; instead, maintaining a domain-specific perspective is essential to effectively address the unique needs of each community.

Regarding responsibility for completing the model card, in research settings it may generally be filled collaboratively by contributors involved in all stages of model development and evaluation. For clinical-grade models, technical specifications (e.g., architecture, software, hardware) could typically be provided by vendors or developers, while clinical teams would rather complete the evaluation and commissioning sections. More formal guidance on stakeholder roles for each section should be further developed in future usability studies.

Another important consideration is the long-term usability of the current model card template. Although we have aimed to design a structure that is robust over time, the rapid evolution of both AI and medical technologies means that some fields may eventually become obsolete, while new ones may need to be introduced. A key open question is how applicable the current version will be to emerging foundation models. As these models demonstrate broader generalization capabilities, the need for certain high-level technical details may diminish, for example, specifying the image field of view or scan parameters may no longer be necessary if the model is designed to handle a wide range of imaging conditions. In this context, ongoing feedback from the community is essential to ensure the model card remains relevant over time. To facilitate this, we have created a dedicated mailing list (rtmodelcard@googlegroups.com) to gather input and suggestions. Furthermore, the model card initiative has been formally adopted as an activity within the ESTRO AI Focus Group, helping to ensure its sustainability and evolution beyond the original authorship.

A standout feature of the proposed model card is Section 4, “Evaluation Data, Methodology, and Results / Commissioning,” which can be repeated per dataset or institution. If such results were shared publicly, this section could serve as a cornerstone for open benchmarking, enabling equitable comparisons of the same model by different groups. However, significant practical challenges exist with the two currently provided formats. On the one hand, the Word-based format limits the potential for scalability and live updates. On the other hand, while the streamlit application enables users to easily add new evaluation data (i.e. by uploading the model card in.json format and adding a new tab in section 4), it does not have hosting capabilities to share this data publicly or through user log-in. A good solution would involve a web-based interface, similar to the ACR AI Lab platform (ailab.acr.org), which claims to provide real-world monitoring of AI models and where participating sites can compare their model’s performance. Comparable efforts in Europe include EUCAIM (https://cancerimage.eu) and, more specifically for radiotherapy, the recently launched dlinrt.eu, a registry of commercial deep learning products that integrates model cards per product. Beyond technical format limitations, the more significant barrier lies in proprietary constraints, as clinics or vendors may be unwilling to publicly disclose commissioning or performance data. Addressing limited institutional disclosure will require coordinated efforts beyond template development. Ongoing initiatives include exploring the integration of the model card within the DLinRT.eu platform, potentially accompanied by an AID-RT–compliant badge to indicate adherence to minimum reporting standards. We are also engaging with the ESTRO working group developing auto-contouring guidelines and with the AAPM Machine Intelligence subcommittee (including TG-384 and TG-424) to promote incorporation of structured model reporting within clinical implementation guidance.

Beyond limiting the potential for open benchmarking, the current Word-based format presents additional usability challenges. Filling in the document manually can be time-consuming, and once completed, the template can easily exceed ten pages, which may lead to information overload. The digital template at https://rt-modelcard.streamlit.app/ tries to overcome these issues, incorporating dropdown menus and standardized fields to streamline the completion process and enable easy export to PDF,.json or markdown (.md). As previously mentioned, the.json format enables users to download and upload the model card to modify or add new information. This app might turn into a valuable asset for our community, since besides standardising and structuring AI documentation, it makes the process more efficient by facilitating information entry. In addition, apart from a few digital templates for generic tools (e.g Tensorflow model card toolkit [37] or Hugging Face Model Card writing tool [32]), this app is the first digital template in the healthcare domain. The usability of this digital template has been tested for three examples of the selected applications, and the result is included in Supplementary Material. Note that these examples are meant to be illustrative, and not serve as a benchmark.

To further improve usability and information rendering, future work could investigate more dynamic, layered formats that present actionable information tailored to user needs. This approach was informed by recent research: for example, a study on model card usability showed that non-experts perceive long documents as less interpretable and less trustworthy [38]. Similarly, Gilbert et al. [39] argued that layered accessible information can enhance real transparency and trust by providing verifiable depth behind summary labels.

Regarding the standardisation of the values in the information fields, we incorporated fixed lists for fields such as anatomical site, input/output content, or dose-engine type, among others, but retained free-text formats for most of the entries to preserve flexibility. Further alignment (e.g., mapping fields to DICOM tags, or expanding controlled vocabularies) could improve interoperability. Yet, excessive rigidity may undermine the model card’s generalisability across diverse RT applications.

It is important to note that our model card emphasized human-readable content, but machine-interpretable documentation is advancing rapidly. Concepts such as the “AI Model Passport”—a standardized machine-readable metadata framework incorporating ontologies and digital identity for models—offer promising paths toward automated tracking and audit compliance [40], [41]. While complementary, these initiatives currently stand apart; integrating machine-readable metadata with our template could significantly bolster reproducibility and traceability in RT.

Finally, industry involvement has already begun, one member from the initial group and multiple reviewers were from RT vendors. We plan to continue this engagement through structured dialogue and pilot collaborations to evaluate the usability and practical implementation of the model card. Continued dialogue with vendors will be crucial for the model card’s evolution, wider adoption, and eventual integration into vendor-hosted platforms or product registries, such as DLinRT.eu. However, broader uptake should not come at the expense of the model card’s core transparency requirements, which should remain driven by clinical and research needs rather than commercial interests.

Our model card template also serves as a valuable educational resource, enhancing AI literacy among clinicians and researchers who may be less familiar with AI models and their evaluation, introducing key concepts in a structured and accessible manner. For instance, the template has recently been integrated into the ESTRO AI course “*Artificial Intelligence in Radiotherapy Clinical Practice*” [42], where participants engaged in a hands-on session comparing two segmentation products using two different model cards. This educational use case highlights the model card’s potential not only as a reporting tool but also as a practical means to foster critical understanding and responsible adoption of AI in radiotherapy.

In conclusion, this work represented the first effort to standardise RT-specific AI model reporting, and led to a consensus-driven model card template that was made publicly available as a Microsoft Word document on Zenodo (https://zenodo.org/records/15336016) and as an interactive digital template at https://rt-modelcard.streamlit.app.

## Declaration of generative AI and AI-assisted technologies in the writing process

During the preparation of this work the author(s) used chatGPT in order to correct grammar and improve the text. After using this tool/service, the author(s) reviewed and edited the content as needed and take(s) full responsibility for the content of the published article.

## Declaration of competing interest

The authors declare the following financial interests/personal relationships which may be considered as potential competing interests: Yvonne de Hond was financially supported by a research grant by Elekta (grant number SOW_20210426, Elekta Ltd., Crawley, UK) until May 2025. The remaining authors declare that they have no known competing financial interests or personal relationships that could have appeared to influence the work reported in this paper.
