# Supplementary material for: AID-RT: Standardising Artificial Intelligence Documentation in RadioTherapy with a domain-specific model card
Source: Phys Imaging Radiat Oncol. 2026 Mar 6;38:100940. doi: 10.1016/j.phro.2026.100940 (PMC12997223; doi:10.1016/j.phro.2026.100940)
Supplement: Supplementary Data 1 [file mmc1.pdf]

Table S1. Positioning of AID-RT with respect to existing AI documentation frameworks. ✓ = fully supported; △ = partially or inconsistently supported; ✗ = not supported. AID-RT uniquely combines domain-specific radiotherapy fields, structured template format, clinical commissioning support, lifecycle traceability, and a deployable digital implementation, addressing limitations of both general-purpose tools and healthcare-specific reporting checklists.

Feature	***General-purpose tools (e.g., Model Cards, Datasheets from Table 1)***	***Healthcare reporting checklists (e.g., CLAIM, TRIPOD+AI from Table 2)***	***AID-RT***
Domain specificity to radiotherapy	*✗ Generic*	*△ Some medical imaging, radiotherapy elements*	***✓ RT-specific fields (e.g., acquisition parameters, dose, segmentation tasks)***
Structured template format	*△ Flexible / narrative*<br>*✓ section based*	*✗ Checklist format (not a template)*<br>*△ Flexible / narrative*<br>*✓ section based*	***✓ Section based, highly structured, granular information fields***
Designed for clinical deployment	*✗ Primarily descriptive, generic*	*✗ Primary descriptive, research reporting focus*	***✓ Includes granular data information (e.g. inclusion criteria for evaluation data) and RT-specific metrics***
Standardised fields for interoperability	*✗ User-defined formatting*	*✗ User-defined formatting*	***✓ Predefined fields enabling comparison (e.g. TG263 compliant)***
Lifecycle traceability (versioning, monitoring)	*△ Limited*	*✗ Not deployment-focused*	***✓ Dedicated metadata and monitoring sections***
Digital implementation	*△ Some platforms (e.g., HuggingFace)*	*✗ No structured tool*	***✓ Streamlit-based structured form***
Target use	*Broad AI transparency*	*Journal reporting / trial reporting*	***Research + clinical implementation in RT***

Table S2. Timeline and details of the model card generation and consensus process.

Phase / Draft	Timeline	Key Activities & Objectives
**First Draft (v0)**	Pre-Sept 2024	Initial template based on Hugging Face fields and participants clinical experience.
**Review Round 1**	Until Sept 27, 2024	Participants proposed edits, additions, removals, and field type changes (required/optional).
**Consolidation**	Late Sept 2024	Group leaders (CB, AB) synthesized feedback and highlighted major/minor suggestions from Round 1.
**Review Round 2**	Until Oct 17, 2024	Voting phase on all comments and field status (Remove, Required, or Optional).
**Consensus Meetings**	Oct 17 & 23, 2024	Two-part meeting to resolve final votes and clarify ambiguous items for re-voting.
**Second Draft (v1)**	Oct 2024	Integration of results from Rounds 1 and 2.
**Review Round 3**	Until Nov 12, 2024	Focused review of highlighted changes from the consensus meetings.
**Third Draft (v2)**	Nov 12, 2024	Updated based on Round 3 votes.
**Fourth Draft (v3)**	Nov 13, 2024	Group leaders (CB,AB) cleaned up the document, and accepted votes by majority. Draft sent for internal/external use-case testing (Synthetic CT, Segmentation, Dose Prediction).
**Refinement**	Dec 13, 2024	Meeting to resolve issues identified during use-case testing; document updated.
**Fifth Draft (v4)**	Jan 27, 2025	Group leaders finalized draft; decision made to seek external/vendor review.
**Sixth Draft (v5)**	Early 2025	Layout adjustments; sent to external reviewers and vendors.
**Review Round 4**	Until Mar 17, 2025	External/Vendor feedback period.
**Seventh Draft (v6)**	Mar 28, 2025	Prepared by leaders to incorporate vendor suggestions for group re-voting.
**Review Round 5**	Until May 23, 2025	Final vote on changes proposed by external stakeholders.
**Final Draft (v7)**	**May 2025**	**Finalized Model Card Template.**

Table S3. Changes suggested by the vendors and accepted by the group. Note that the group accepted to make optional the fields regarding the learning architecture (inputs/outputs) since the section “Model overview” contains required (high-level) information about the model inputs and outputs that were deemed sufficient to have enough information about the model pipeline.

**Type of information**	**Specific information fields affected**	**Change applied**
Model card metadata	● Creation date	Clarification of the definition
Contact information	● Approved by / Name<br>● Approved by / Contact email<br>● Developed by / Name<br>● Developed by / Contact email	Changed from required to optional
Learning architecture	● Number of inputs<br>● Input content<br>● Input size<br>● Number of output<br>● Output content<br>● Output size	Changed from required to optional
Training methodology	● Type of data augmentation<br>● Strategy for data augmentation	Changed from required to optional
