# Supplementary material for: AID-RT: Standardising Artificial Intelligence Documentation in RadioTherapy with a domain-specific model card
Source: Phys Imaging Radiat Oncol. 2026 Mar 6;38:100940. doi: 10.1016/j.phro.2026.100940 (PMC12997223; doi:10.1016/j.phro.2026.100940)
Supplement: Supplementary Data 2 [file mmc2.pdf]

# Model Card — version 1.0 — Model sCT Toolbox

**Task:** Image-to-Image translation

---

## 0. Card Metadata

**Creation date**: 2025/08/26

### Versioning

- **Version number**: 1.0
- **Version changes**: version 1.0

---

## 1. Model Basic Information

**Name**: sCT Toolbox

**Creation date**: 2023/01/23

### Versioning

- **Version number**: 01.00.0000
- **Version changes**: None

### Model scope

- **Summary**: CBCT-based Synthetic CT Generation Model
- **Anatomical site**: HN

### Clearance

- **Type**: Not approved for medical use - research only

#### Approved by

- **Name(s)**: Arthur Galapon
- **Institution(s)**: University Medical Center Groningen
- **Contact email(s)**: a.v.galapon@umcg.nl

**Intended users**: Medical Physicists, Dosimetrists, RTTs

**Observed limitations**: Not applicable to images before Adapt Insight v2.2

**Potential limitations**: CBCT images with open air gaps (lung) due to limited FoV

**Type of learning architecture**: UNET

### Developed by

- **Name**: Arthur Galapon
- **Institution(s)**: UMC Groningen
- **Contact email(s)**: a.v.galapon@umcg.nl

**Conflict of interest**: NA

**Software licence**: NA

**Code source**: NA

**Model source**: NA

**Citation details**: NA

**URL info**: NA

---

# 2. Technical specifications

## 2.1 Model overview

### Model pipeline

- **Summary:** CBCT images are resampled prior to being processed by the model to generate synthetic CTs. Post-processing involves extraction of the reference frame from the input CBCT
- **Model inputs:** ['CBCT', 'Binary mask']
- **Model outputs:** ['CT']
- **Pre-processing:** Conversion to NRRD/NIFTI, Resampling, Mask Segmentation
- **Post-processing:** Conversion to DICOM

## 2.2 Learning architecture(s)

### Learning architecture 1

| Field | Value |
|---|---|
| **Total number of trainable parameters** | 50351810 |
| **Number of inputs** | 1 |
| **Input content** | — |
| **Additional information regarding input content** | NA |
| **Input format** | [array, array] |
| **Input size** | [[1,512,512],[1,512,512]] |
| **Number of outputs** | 1 |

| Field | Value |
|---|---|
| **Output content** | — |
| **Additional information regarding output content** | NA |
| **Output format** | [array] |
| **Output size** | [1,512,512] |
| **Loss function** | L1 |
| **Batch size** | 1 |
| **Regularisation** | Dropout |
| **Architecture figure** | learning_architecture_0_architecture_figure_DCNN-diagram.png |

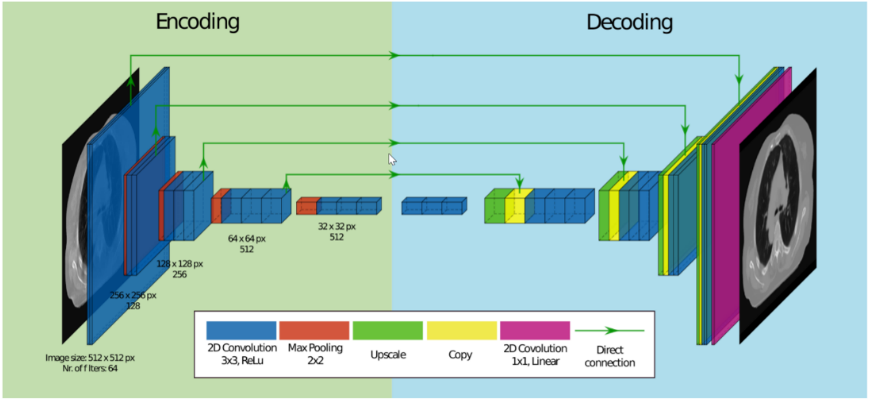


| | **Uncertainty quantification techniques** | Monte Carlo at T=10 | | **Explainability techniques** | NA | | **Additional info** | NA | | **Citation(s)** | NA |

## 2.3 Hardware & software

- **Libraries and dependencies:** Pytorch 2.0.1, SimpleITK 2.0.0
- **Hardware (recommended):** at leaset 46GB A40 GPU
- **Inference time (s) for recommended hardware:** 240.0
- **Installation / Getting started:** NA
- **Environmental impact:** NA

---

# 3. Training Data Methodology and Information

### Fine tuned form

- **Model name:** NA
- **URL/DOI to model card:** NA
- **Tuning technique:** NA

## Training Dataset

### General information

- **Total size:** [14250]
- **Number of patients:** 57
- **Source:** Private dataset from clinic
- **Acquisition period:** December 2022 - August 2023
- **Inclusion / exclusion criteria:** Patients with same-day rCT and CBCT
- **Type of data augmentation:** Flipping [left-right, top-down], Shifting [x-direction, y-direction, diagonal]
- **Strategy for data augmentation:** [random, on-the-fly during training]
- **URL info:** NA

### Technical specifications

**CBCT** (model_inputs)

| Field | Value |
|---|---|
| **Image resolution** | [0.65,0.65,2.5] |
| **Patient positioning** | [Head First Supine] |
| **Scan(s) manufacturer and model** | Proteus Plus Gantry CBCT |
| **Scan acquisition parameters** | [CT:120kV] |
| **Scan reconstruction parameters** | AdaptInsight |
| **FOV** | [500,500,350] |

**Binary mask** (model_inputs)

| Field | Value |
|---|---|
| **Image resolution** | [1,1,1] |
| **Patient positioning** | NA |
| **Scan(s) manufacturer and model** | NA |
| **Scan acquisition parameters** | NA |
| **Scan reconstruction parameters** | NA |
| **FOV** | NA |

**CT** (model_outputs)

| Field | Value |
| --- | --- |
| **Image resolution** | [0.976,0.976,2.0] |
| **Patient positioning** | [Head First Supine] |
| **Scan(s) manufacturer and model** | [Siemens Somatom Confidence] |
| **Scan acquisition parameters** | [CT:120kVp] |
| **Scan reconstruction parameters** | QR40s |
| **FOV** | [500,500,392] |

- **Reference standard:** NA
- **Reference standard QA:** NA
- **Additional information:** NA

### Patient demographics and clinical characteristics

- **ICD10/11:** NA
- **TNM staging:** NA
- **Age:** NA
- **Sex:** NA
- **Target volume (cm³):** NA
- **BMI:** NA
- **Additional patient info:** NA **Validation strategy:** Use of Validation set

**Validation data partition:** [20%]

**Weights initialization:** Uniform **Epochs:** [200] **Optimiser:** Adam **Learning rate:** 1e-5

**Model choice criteria:** Early stopping based on validation

**Inference method:** average at T=10 inferences

---

# 4. Evaluation Data Methodology, Results and Commissioning

## 1 Evaluation

**Evaluation date:** 2022/08/01

### Evaluated by

- **Name(s):** Arthur Galapon
- **Institution(s):** University Medical Center Groningen
- **Contact email(s):** a.v.galapon@umcg.nl
- **Same as 'Approved by':** Yes

**Evaluation frame:** Quantitative

**Sanity check:** Resulting sCT is cross-checked to its ground truth; validating correct HU range.

## Evaluation dataset

### General information

- **Total size:** [21]
- **Number of patients:** 21
- **Source:** Private dataset from clinic
- **Acquisition period:** December 2022 - August 2023
- **Inclusion / Exclusion criteria:** patients with same-day rCT and CBCT
- **URL info:** NA

### Technical specifications

**CBCT** (model_inputs)

| Field | Value |
|---|---|
| **Image resolution** | [0.65,0.65,2.5] |
| **Patient positioning** | [Head First Supine] |
| **Scan(s) manufacturer and model** | Proteus Plus Gantry CBCT |
| **Scan acquisition parameters** | [CT:120kV] |
| **Scan reconstruction parameters** | AdaptInsight |
| **FOV** | [500,500,350] |

**Binary mask** (model_inputs)

| Field | Value |
|---|---|
| **Image resolution** | [1,1,1] |
| **Patient positioning** | NA |
| **Scan(s) manufacturer and model** | NA |
| **Scan acquisition parameters** | NA |
| **Scan reconstruction parameters** | NA |
| **FOV** | NA |

**CT** (model_outputs)

| Field | Value |
|---|---|
| **Image resolution** | [0.976,0.976,2.0] |

| Field | Value |
| --- | --- |
| **Patient positioning** | [Head First Supine] |
| **Scan(s) manufacturer and model** | [Siemens Somatom Confidence] |
| **Scan acquisition parameters** | [CT:120kVp] |
| **Scan reconstruction parameters** | QR40s |
| **FOV** | [500,500,392] |

- **Reference standard:** NA
- **Reference standard QA:** NA
- **Additional information:** NA

### Patient demographics and clinical characteristics

- **ICD10/11:** NA
- **TNM staging:** NA
- **Age:** NA
- **Sex:** NA
- **Target volume (cm³):** NA
- **BMI:** NA
- **Additional information:** NA

## Quantitative evaluation

### Image Similarity Metrics

#### MAE (Mean Absolute Error)

| Field | Value |
| --- | --- |
| Type | MAE (Mean Absolute Error) |
| On Volume | Body |
| Registration | NONRIGID |
| Sample Data | NA |
| Mean Data | [81.81, 65.61, 177.26, 10.51] |
| Figure Appendix Label | — |

#### SSIM (Structural Similarity Index)

| Field | Value |
|---|---|
| Type | SSIM (Structural Similarity Index) |
| On Volume | Body |
| Registration | NONRIGID |
| Sample Data | NA |
| Mean Data | [0.982, 0.956, 0.991, 0.006] |
| Figure Appendix Label | — |

**PSNR (Peak Signal-to-Noise Ratio)**

| Field | Value |
|---|---|
| Type | PSNR (Peak Signal-to-Noise Ratio) |
| On Volume | Body |
| Registration | NONRIGID |
| Sample Data | NA |
| Mean Data | [27.27, 19.52, 29.57, 1.13] |
| Figure Appendix Label | — |

**Figure MAE (Mean Absolute Error)**

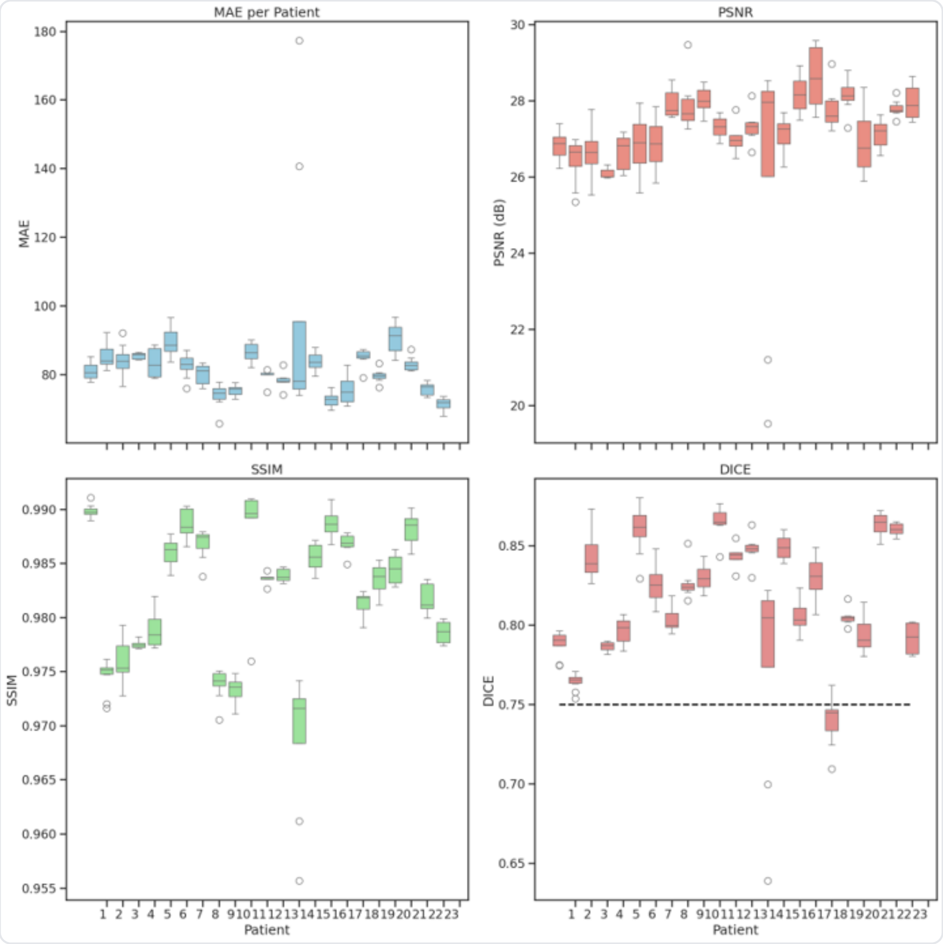


*Appendix label:* —

## Dose Metrics

### GPR (Gamma Passing Rate)

| Field | Value |
|---|---|
| Type | GPR (Gamma Passing Rate) |
| Metric Specifications | 2%/2mm |
| On Volume | Body |
| Registration | NONRIGID |
| Treatment Modality | External beam radiation therapy (EBRT) - Protons - Scanning beam multi-field optimization |
| Dose Engine | Monte Carlo |
| Dose Grid Resolution | [3,3,3] |
| TPS Vendor | Raystation |
| Sample Data | NA |
| Mean Data | [97.39, 89.57, 99.51, 2.51] |

| Field | Value |
| --- | --- |
| Figure Appendix Label | — |

### Qualitative evaluation

**Evaluators information:** NA

**Likert scoring**

- Method: NA
- Results: NA

**Turing test**

- Method: NA
- Results: NA

**Time saving**

- Method: NA
- Results: NA

**Other**

- Method: NA
- Results: NA

**Explainability:** NA

**Citation details:** NA

---

## 5. Other considerations

- **Responsible Use and Ethical Considerations:** NA
- **Risk Analysis:** NA
- **Post-Market Surveillance / Live Monitoring:** NA

---
