# Supplementary material for: AID-RT: Standardising Artificial Intelligence Documentation in RadioTherapy with a domain-specific model card
Source: Phys Imaging Radiat Oncol. 2026 Mar 6;38:100940. doi: 10.1016/j.phro.2026.100940 (PMC12997223; doi:10.1016/j.phro.2026.100940)

Model Card — version 1 — Model Task618_WorkShopLeftSidedAllLymphnodes_07_11

Task: Segmentation

---

0. Card Metadata

Creation date: 2025/09/04

Versioning

- **Version number**: 1
- **Version changes**: NA

---

1. Model Basic Information

Name: Task618_WorkShopLeftSidedAllLymphnodes_07_11

Creation date: 2023/11/07

Versioning

- **Version number**: 01.00.0000
- **Version changes**: NA

Model scope

- **Summary**: Auto-segmentation model of left-sided breast cancer lymph node levels
- **Anatomical site**: Thorax

Clearance

- **Type**: Not approved for medical use - only research use

Approved by

- **Name(s)**: NA
- **Institution(s)**: NA
- **Contact email(s)**: NA

Intended users: Delineators (Radiation oncologists or radiotherapists)

Observed limitations: Air-cavities interfering with normal location of lymph nodes

Potential limitations: Implants, large seromas, non-supine positions, other fixation types

Type of learning architecture: UNet (used nn-Unet version 1)

Developed by

- **Name**: Emma Skarsø Buhl, on behalf of the Danish Breast Cancer Group RT
- **Institution(s)**: Aarhus University Hospital, Odense University Hospital, University Hospital of Southern Denmark, Vejle Hospital, Aalborg University Hospital, Zealand University Hospital, Copenhagen University Hospital - Rigshospitalet, Copenhagen University Hospital - Herlev and Gentofte
- **Contact email(s)**: emskar@rm.dk, stine.korreman@clin.au.dk

Conflict of interest: NA

Software licence: NA

Code source: NA

Model source: NA

Citation details: Skarsø EB, Lorenzen EL, Refsgaard L, Nielsen AWM, Brixen ATL, Maae E, Holm HS, Schøler J, Thai LMH, Matthiessen LW, Maraldo MV, Nielsen MM, Johansen MB, Milo ML, Mogensen MB, Nielsen MH, Møller M, Sand M, Schultz P, Al-Rawi, SAJ, Esser-Naumann S, Yammeni S, Petersen SE, Offersen BV, Korreman SS. Development and comprehensive evaluation of a national DBCG consensusbased auto-segmentation model for lymph node levels in breast cancer radiotherapy. Radiotherapy and Oncology 2024;201. https:// doi.org/10.1016/j.radonc.2024.110567

URL info: https://www.sciencedirect.com/science/article/pii/S016781402403545X?via%3Dihub

---

2. Technical specifications

2.1 Model overview

Model pipeline

- **Summary:** A CT scan is sent to a 3D nnU-Net, which outputs NIfTI files that are later converted into DICOM RTSTRUCT.
- **Model inputs:** ['CT']
- **Model outputs:** ['RTSTRUCT_CTVn_L1', 'RTSTRUCT_CTVn_L2', 'RTSTRUCT_CTVn_L3', 'RTSTRUCT_CTVn_L4', 'RTSTRUCT_CTVn_IMN_IC3', 'RTSTRUCT_CTVn_Interpect']
- **Pre-processing:** NA
- **Post-processing:** If overlap between the CTVn_IMN and the CTVn_L4, the overlap from the CTVn_IMN were added to the CTVn_L4. If any additional subvolumes appeared other places in the body, they were removed. If the most cranial and/or caudal slice was 10% of the volume of the previous slice, they were removed. NIfTI output was converted into DICOM RTSTRUCT using a modified version of rt-utils

2.2 Learning architecture(s)

Learning architecture 1

Field	Value
**Total number of trainable parameters**	NA
**Number of inputs**	1

Field	Value
**Input content**	—
**Input format**	NIfTI
**Input size**	NA
**Number of outputs**	1
**Output content**	—
**Output format**	NIfTI
**Output size**	NA
**Loss function**	MDice + Cross entropy (nnU-Net default)
**Batch size**	2
**Regularisation**	—
**Uncertainty quantification techniques**	NA
**Explainability techniques**	NA
**Additional info**	nnU-Net default settings have been used
**Citation(s)**	Isensee, F., Jaeger, P.F., Kohl, S.A.A. et al. nnU-Net: a self-configuring method for deep learning-based biomedical image segmentation. Nat Methods 18, 203–211 (2021). https://doi.org/10.1038/s41592-020-01008-z

2.3 Hardware & software

No hardware and software details specified.

---

3. Training Data Methodology and Information

Fine tuned form

- **Model name:** NA
- **URL/DOI to model card:** NA
- **Tuning technique:** NA

Training Dataset

General information

- **Total size:** [122]

- **Number of patients:** 122
- **Source:** Private dataset owned by the Danish Breast Cancer Group RT from all seven RT centres in Denmark (University Hospital of Southern Denmark - Vejle Hospital, Aarhus University Hospital, Aalborg University Hospital, Odense University Hospital, Zealand University Hospital - Næstved Hospital, Copenhagen University Hospital - Rigshospitalet and Copenhagen University Hospital - Herlev Hospital)
- **Acquisition period:** The patients in the dataset were treated in the period from January 2015-December 2016. The delineations on the patients were corrected according to the ESTRO delineation guidelines during October 2022-January 2023. The delineations were corrected by delineation experts from all radiotherapy and proton therapy centres in Denmark.
- **Inclusion / exclusion criteria:** Patients were chosen to be evenly distributed between treating centre. Patients with most clinically delineated target structures were prioritized. Patients with bilateral breast cancer were excluded.
- **Type of data augmentation:** Rotations, scaling, Gaussian noise, Gaussian blur, brightness, contrast, simulation of low-resolution, gamma augmentation and mirroring (standard data augmentation used by default in nnU-Net).
- **Strategy for data augmentation:** [on-the-fly during training, most of the augmentations are varied with parameters drawn randomly from predefined ranges, all augmentations are applied stochastically according to a predefined probability]
- **URL info:** For more information about dataset generation, see publication: 10.1016/j.radonc.2024.110567

Technical specifications

CT (model_inputs)

Field	Value
**Image resolution**	Pixel size: 0.98mm [0.75mm-1.37mm], slice thickness: 2.5mm [2.0mm-3.0mm], CT length: 376mm [307.5mm-524.0mm]
**Patient positioning**	[Supine, with either both arms or one arm elevated]
**Scan(s) manufacturer and model**	[Philips, GE Medical Systems, Siemens]
**Scan acquisition parameters**	[GE Medical Systems:120kV, Philips:120kV,140kV, Siemens:100kV, 120 kV, 140 kV]
**Scan reconstruction parameters**	NA
**FOV**	NA

RTSTRUCT_CTVn_L1 (model_outputs)

Field	Value
**Image resolution**	Pixel size: 0.98mm [0.75mm-1.37mm], slice thickness: 2.5mm [2.0mm-3.0mm], CT length: 376mm [307.5mm-524.0mm]
**Patient positioning**	[Supine, with either both arms or one arm elevated]

Field	Value
**Scan(s) manufacturer and model**	NA
**Scan acquisition parameters**	NA
**Scan reconstruction parameters**	NA
**FOV**	NA

RTSTRUCT_CTVn_L2 (model_outputs)

Field	Value
**Image resolution**	Pixel size: 0.98mm [0.75mm-1.37mm], slice thickness: 2.5mm [2.0mm-3.0mm], CT length: 376mm [307.5mm-524.0mm]
**Patient positioning**	[Supine, with either both arms or one arm elevated]
**Scan(s) manufacturer and model**	NA
**Scan acquisition parameters**	NA
**Scan reconstruction parameters**	NA
**FOV**	NA

RTSTRUCT_CTVn_L3 (model_outputs)

Field	Value
**Image resolution**	Pixel size: 0.98mm [0.75mm-1.37mm], slice thickness: 2.5mm [2.0mm-3.0mm], CT length: 376mm [307.5mm-524.0mm]
**Patient positioning**	[Supine, with either both arms or one arm elevated]
**Scan(s) manufacturer and model**	NA
**Scan acquisition parameters**	NA
**Scan reconstruction parameters**	NA
**FOV**	NA

RTSTRUCT_CTVn_L4 (model_outputs)

Field	Value
**Image resolution**	Pixel size: 0.98mm [0.75mm-1.37mm], slice thickness: 2.5mm [2.0mm-3.0mm], CT length: 376mm [307.5mm-524.0mm]
**Patient positioning**	[Supine, with either both arms or one arm elevated]

Field	Value
**Scan(s) manufacturer and model**	NA
**Scan acquisition parameters**	NA
**Scan reconstruction parameters**	NA
**FOV**	NA

RTSTRUCT_CTVn_IMN_IC3 (model_outputs)

Field	Value
**Image resolution**	Pixel size: 0.98mm [0.75mm-1.37mm], slice thickness: 2.5mm [2.0mm-3.0mm], CT length: 376mm [307.5mm-524.0mm]
**Patient positioning**	[Supine, with either both arms or one arm elevated]
**Scan(s) manufacturer and model**	NA
**Scan acquisition parameters**	NA
**Scan reconstruction parameters**	NA
**FOV**	NA

RTSTRUCT_CTVn_Interpect (model_outputs)

Field	Value
**Image resolution**	Pixel size: 0.98mm [0.75mm-1.37mm], slice thickness: 2.5mm [2.0mm-3.0mm], CT length: 376mm [307.5mm-524.0mm]
**Patient positioning**	[Supine, with either both arms or one arm elevated]
**Scan(s) manufacturer and model**	NA
**Scan acquisition parameters**	NA
**Scan reconstruction parameters**	NA
**FOV**	NA

- **Reference standard:** ESTRO consensus delineation guideline on target volume delineation of early-stage breast cancer, BV Offersen et al, doi:10.1016/j.radonc.2014.11.030
- **Reference standard QA:** The dataset generation was started during a national target delineation workshop. The participants were breast cancer delineation experts from the whole nation (n=21). The workshop was initiated with a discussion of the interpretations of the delineation guidelines used nationally. Following that a gold standard dataset was generated with five observer delineation on each patient (20 in total). After the gold standard dataset generation, the participants received patients (treated in the seven radiotherapy centres in 2015-2016) with existing clinical target delineations used to treat the patients. The participants were asked to correct the existing

target delineations according to the interpretations discussed at the workshop. For more details see the publication: 10.1016/j.radonc.2024.110567

Patient demographics and clinical characteristics

- **Age:** [58,49-66]
- **Sex:** 100%F
- **Target volume (cm³):** [CTVn_L1 = 46.1,18.2-140],[CTVn_L2 = 18.5,7.71-49.4], [CTVn_L3 = 13.2,6.13-31.8], [CTVn_L4 = 13.1, 6.31-31.2], [CTVn_IMN = 9.4, 2.87-21.1], [CTVn_Interpect = 12.8, 4.07-43.6]
- **Additional patient info:** For more information about patient population, see following publications: 10.1016/j.radonc.2024.110289 and 10.1016/j.phro.2023.100485 **Validation strategy:** 5-fold cross-validation

Validation data partition: [20%, 20%, 20%, 20%, 20%]

Epochs: [300, 300, 300, 300, 300] **Optimiser:** SGD (nnU-Net default) **Learning rate:** 0.00997

Model choice criteria: Last epoch

Inference method: 5-fold ensemble

---

4. Evaluation Data Methodology, Results and Commissioning

1 Evaluation in hold-out test set

Evaluation date: 2024/01/01

Evaluated by

- **Name(s):** Emma Skarsø Buhl
- **Institution(s):** Aarhus University Hospital
- **Contact email(s):** emskar@rm.dk
- **Same as 'Approved by':** No

Evaluation frame: Retrospective evaluation with hold-out test set from the dataset pool used to train the model

Evaluation dataset

General information

- **Total size:** [13]
- **Number of patients:** 13
- **Source:** Private dataset owned by the Danish Breast Cancer Group RT from all seven RT centres in Denmark (University Hospital of Southern Denmark - Vejle Hospital, Aarhus University Hospital, Aalborg University Hospital, Odense University Hospital, Zealand University Hospital - Næstved Hospital, Copenhagen University Hospital - Rigshospitalet and Copenhagen University Hospital - Herlev Hospital)
- **Acquisition period:** The patients in the dataset were treated in the period from January 2015-December 2016. The delineations on the patients were corrected according to the ESTRO delineation guidelines during October

2022-January 2023. The delineations were corrected by delineation experts from all radiotherapy and proton therapy centres in Denmark.

- **Inclusion / Exclusion criteria:** Patients were chosen to be evenly distributed between treating centre. Patients with most clinically delineated target structures were prioritized. Patients with bilateral breast cancer were excluded.
- **URL info:** For more information about dataset generation, see publication: 10.1016/j.radonc.2024.110567

Technical specifications

CT (model_inputs)

Field	Value
**Image resolution**	Pixel size: 0.98mm [0.75mm-1.37mm], slice thickness: 2.5mm [2.0mm-3.0mm], CT length: 376mm [307.5mm-524.0mm]
**Patient positioning**	[Supine, with either both arms or one arm elevated]
**Scan(s) manufacturer and model**	[Philips, GE Medical Systems, Siemens]
**Scan acquisition parameters**	[Philips: 120kV, GE Medical Systems: 120 kV, Siemens: 100kV,120 kV]
**Scan reconstruction parameters**	NA
**FOV**	NA

RTSTRUCT_CTVn_L1 (model_outputs)

Field	Value
**Image resolution**	Pixel size: 0.98mm [0.75mm-1.37mm], slice thickness: 2.5mm [2.0mm-3.0mm], CT length: 376mm [307.5mm-524.0mm]
**Patient positioning**	[Supine, with either both arms or one arm elevated]
**Scan(s) manufacturer and model**	NA
**Scan acquisition parameters**	NA
**Scan reconstruction parameters**	NA
**FOV**	NA

RTSTRUCT_CTVn_L2 (model_outputs)

Field	Value
**Image resolution**	Pixel size: 0.98mm [0.75mm-1.37mm], slice thickness: 2.5mm [2.0mm-3.0mm], CT length: 376mm [307.5mm-524.0mm]

Field	Value
**Patient positioning**	[Supine, with either both arms or one arm elevated]
**Scan(s) manufacturer and model**	NA
**Scan acquisition parameters**	NA
**Scan reconstruction parameters**	NA
**FOV**	NA

RTSTRUCT_CTVn_L3 (model_outputs)

Field	Value
**Image resolution**	Pixel size: 0.98mm [0.75mm-1.37mm], slice thickness: 2.5mm [2.0mm-3.0mm], CT length: 376mm [307.5mm-524.0mm]
**Patient positioning**	[Supine, with either both arms or one arm elevated]
**Scan(s) manufacturer and model**	NA
**Scan acquisition parameters**	NA
**Scan reconstruction parameters**	NA
**FOV**	NA

RTSTRUCT_CTVn_L4 (model_outputs)

Field	Value
**Image resolution**	Pixel size: 0.98mm [0.75mm-1.37mm], slice thickness: 2.5mm [2.0mm-3.0mm], CT length: 376mm [307.5mm-524.0mm]
**Patient positioning**	[Supine, with either both arms or one arm elevated]
**Scan(s) manufacturer and model**	NA
**Scan acquisition parameters**	NA
**Scan reconstruction parameters**	NA
**FOV**	NA

RTSTRUCT_CTVn_IMN_IC3 (model_outputs)

Field	Value
**Image resolution**	Pixel size: 0.98mm [0.75mm-1.37mm], slice thickness: 2.5mm [2.0mm-3.0mm], CT length: 376mm [307.5mm-524.0mm]

Field	Value
**Patient positioning**	[Supine, with either both arms or one arm elevated]
**Scan(s) manufacturer and model**	NA
**Scan acquisition parameters**	NA
**Scan reconstruction parameters**	NA
**FOV**	NA

RTSTRUCT_CTVn_Interpect (model_outputs)

Field	Value
**Image resolution**	Pixel size: 0.98mm [0.75mm-1.37mm], slice thickness: 2.5mm [2.0mm-3.0mm], CT length: 376mm [307.5mm-524.0mm]
**Patient positioning**	[Supine, with either both arms or one arm elevated]
**Scan(s) manufacturer and model**	NA
**Scan acquisition parameters**	NA
**Scan reconstruction parameters**	NA
**FOV**	NA

- **Reference standard:** ESTRO consensus delineation guideline on target volume delineation of early-stage breast cancer, BV Offersen et al, doi:10.1016/j.radonc.2014.11.030
- **Reference standard QA:** The dataset generation was started during a national target delineation workshop. The participants were breast cancer delineation experts from the whole nation (n=21). The workshop was initiated with a discussion of the interpretations of the delineation guidelines used nationally. Following that a gold standard dataset was generated with five observer delineation on each patient (20 in total). After the gold standard dataset generation, the participants received patients (treated in the seven radiotherapy centres in 2015-2016) with existing clinical target delineations used to treat the patients. The participants were asked to correct the existing target delineations according to the interpretations discussed at the workshop. The dataset used to evaluated was a hold-out dataset from the training dataset.
- **Additional information:** For more details see the publication: 10.1016/j.radonc.2024.110567

Patient demographics and clinical characteristics

- **Age:** [58,49-66]
- **Sex:** 100%F
- **Target volume (cm³):** [CTVn_L1 = 51.3,13.7-77.7],[CTVn_L2 = 18.7,8.88-30.0], [CTVn_L3 = 11.2,5.19-21.6], [CTVn_L4 = 12.8, 6.71-19.6], [CTVn_IMN = 8.12, 3.17-12.5], [CTVn_Interpect = 12.8, 4.60-27.3]

Quantitative evaluation

Geometric Metrics

DSC (Dice Similarity Coefficient) CTVn_L1

Field	Value
Type	DSC (Dice Similarity Coefficient) CTVn_L1
Metric Specifications	Python MedPy package
On Volume	CTVn_L1
Sample Data	[0.88, 0.87, 0.83, 0.68, 0.80, 0.89, 0.80, 0.70, 0.77, 0.67, 0.86, 0.45, 0.88]
Mean Data	[0.77, 0.45, 0.89, 0.12]
Figure Appendix Label	—

DSC (Dice Similarity Coefficient) CTVn_L2

Field	Value
Type	DSC (Dice Similarity Coefficient) CTVn_L2
Metric Specifications	Python MedPy package
On Volume	CTVn_L2
Sample Data	[0.85, 0.88, 0.84, 0.80, 0.86, 0.86, 0.82, 0.74, 0.77, 0.73, 0.87, 0.84, 0.90]
Mean Data	[0.83, 0.73, 0.90, 0.05]
Figure Appendix Label	—

DSC (Dice Similarity Coefficient) CTVn_L3

Field	Value
Type	DSC (Dice Similarity Coefficient) CTVn_L3
Metric Specifications	Python MedPy package
On Volume	CTVn_L3
Sample Data	[0.90, 0.84, 0.89, 0.89, 0.88, 0.87, 0.84, 0.74, 0.73, 0.74, 0.89, 0.90, 0.91]
Mean Data	[0.85, 0.73, 0.91, 0.07]
Figure Appendix Label	—

DSC (Dice Similarity Coefficient) CTVn_L4

Field	Value
Type	DSC (Dice Similarity Coefficient) CTVn_L4
Metric Specifications	Python MedPy package
On Volume	CTVn_L4
Sample Data	[0.90, 0.82, 0.93, 0.82, 0.85, 0.86, 0.82, 0.60, 0.76, 0.70, 0.89, 0.81, 0.94]
Mean Data	[0.82, 0.60, 0.94, 0.09]
Figure Appendix Label	—

DSC (Dice Similarity Coefficient) CTVn_IMN_IC3

Field	Value
Type	DSC (Dice Similarity Coefficient) CTVn_IMN_IC3
Metric Specifications	Python MedPy package
On Volume	CTVn_IMN_IC3
Sample Data	[0.84, 0.84, 0.80, 0.77, 0.81, 0.80, 0.78, 0.78, 0.64, 0.81, 0.79, 0.80, 0.87]
Mean Data	[0.75, 0.56, 0.87, 0.09]
Figure Appendix Label	—

DSC (Dice Similarity Coefficient) CTVn_Interpect

Field	Value
Type	DSC (Dice Similarity Coefficient) CTVn_Interpect
Metric Specifications	Python MedPy package
On Volume	CTVn_Interpect
Sample Data	[0.72, 0.87, 0.80, 0.78, 0.56, 0.82, 0.82, 0.74, 0.64, 0.71, 0.83, 0.72, 0.82]
Mean Data	[0.79, 0.64, 0.87, 0.05]
Figure Appendix Label	—

HD95 (95th Percentile Hausdorff Distance) CTVn_L1

Field	Value
Type	HD95 (95th Percentile Hausdorff Distance) CTVn_L1
Metric Specifications	Python MedPy package

Field	Value
On Volume	CTVn_L1
Sample Data	[4.32, 4.03, 4.78, 24.0, 5.13, 4.00, 6.54, 12.3, 12.0, 8.98, 5.31, 27.5, 6.08]
Mean Data	[9.62, 4.00, 27.5, 7.73]
Figure Appendix Label	—

HD95 (95th Percentile Hausdorff Distance) CTVn_L2

Field	Value
Type	HD95 (95th Percentile Hausdorff Distance) CTVn_L2
Metric Specifications	Python MedPy package
On Volume	CTVn_L2
Sample Data	[2.76, 3.00, 2.66, 3.00, 2.47, 2.43, 2.29, 7.28, 4.77, 5.93, 2.75, 4.06, 2.18]
Mean Data	[3.51, 2.18, 7.28, 1.58]
Figure Appendix Label	—

HD95 (95th Percentile Hausdorff Distance) CTVn_L3

Field	Value
Type	HD95 (95th Percentile Hausdorff Distance) CTVn_L3
Metric Specifications	Python MedPy package
On Volume	CTVn_L3
Sample Data	[2.50, 3.00, 2.50, 1.66, 2.05, 2.23, 2.25, 4.56, 4.69, 6.04, 2.18, 2.50, 3.00]
Mean Data	[3.01, 1.66, 6.04, 1.28]
Figure Appendix Label	—

HD95 (95th Percentile Hausdorff Distance) CTVn_L4

Field	Value
Type	HD95 (95th Percentile Hausdorff Distance) CTVn_L4
Metric Specifications	Python MedPy package
On Volume	CTVn_L4
Sample Data	

Field	Value
	[2.68, 3.00, 1.29, 3.81, 2.90, 2.76, 2.24, 11.5, 6.22, 7.36, 2.23, 4.43, 1.38]
Mean Data	[3.99, 1.29, 11.5, 2.86]
Figure Appendix Label	—

HD95 (95th Percentile Hausdorff Distance) CTVn_IMN_IC3

Field	Value
Type	HD95 (95th Percentile Hausdorff Distance) CTVn_IMN_IC3
Metric Specifications	Python MedPy package
On Volume	CTVn_IMN_IC3
Sample Data	[2.50, 2.18, 2.66, 3.00, 2.05, 2.23, 2.47, 13.3, 10.7, 6.61, 2.43, 7.55, 1.95]
Mean Data	[4.27, 1.95, 22.0, 5.40]
Figure Appendix Label	—

HD95 (95th Percentile Hausdorff Distance) CTVn_Interpect

Field	Value
Type	HD95 (95th Percentile Hausdorff Distance) CTVn_Interpect
Metric Specifications	Python MedPy package
On Volume	CTVn_Interpect
Sample Data	[2.68, 1.95, 2.50, 2.62, 22.0, 2.18, 2.05, 3.56, 4.77, 3.68, 1.95, 3.55, 1.95]
Mean Data	[4.59, 1.95, 13.3, 3.78]
Figure Appendix Label	—

Qualitative evaluation

Evaluators information: —

Likert scoring

- Method: —
- Results: —

Turing test

- Method: —

- Results: —

Time saving

- Method: —
- Results: —

Other

- Method: —
- Results: —

Explainability: —

Citation details: —

---

5. Other considerations

No other considerations provided.

Appendix

progress_fold4.png

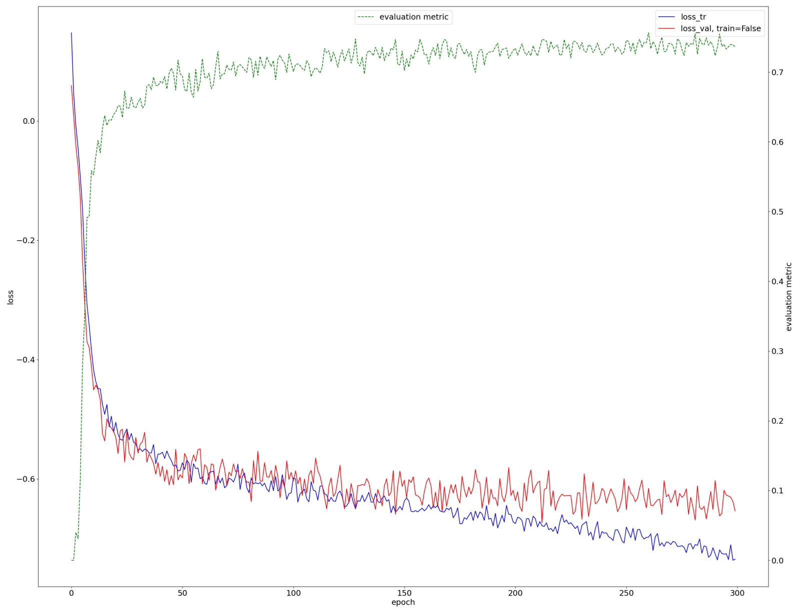

progress_fold3.png

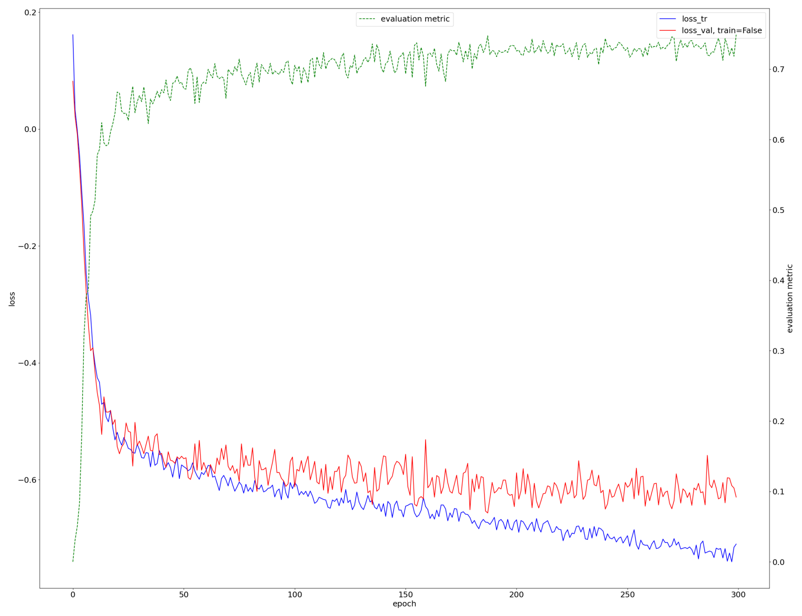

progress_fold2.png

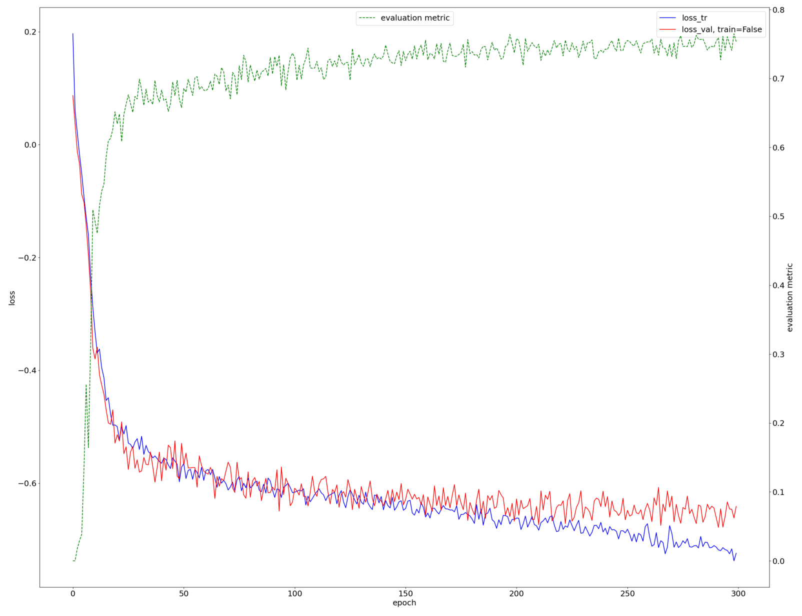

progress_fold1.png

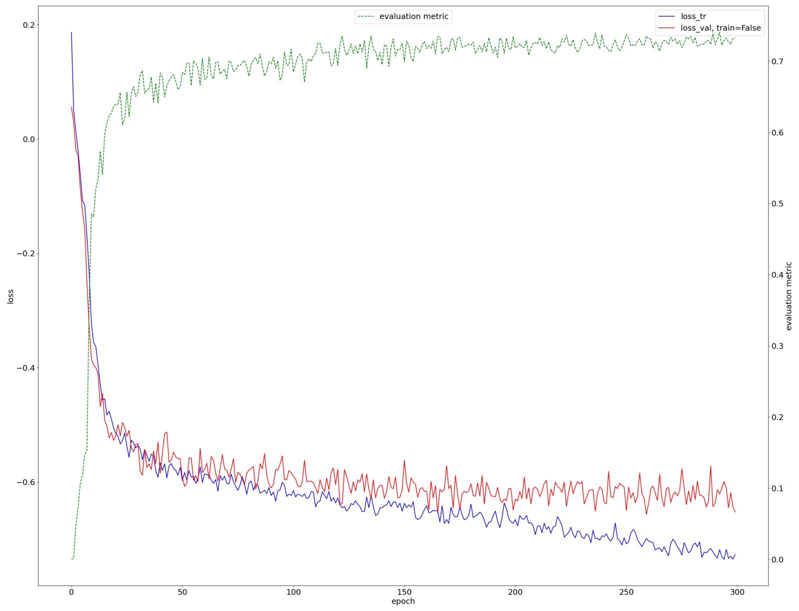

progress_fold0.png

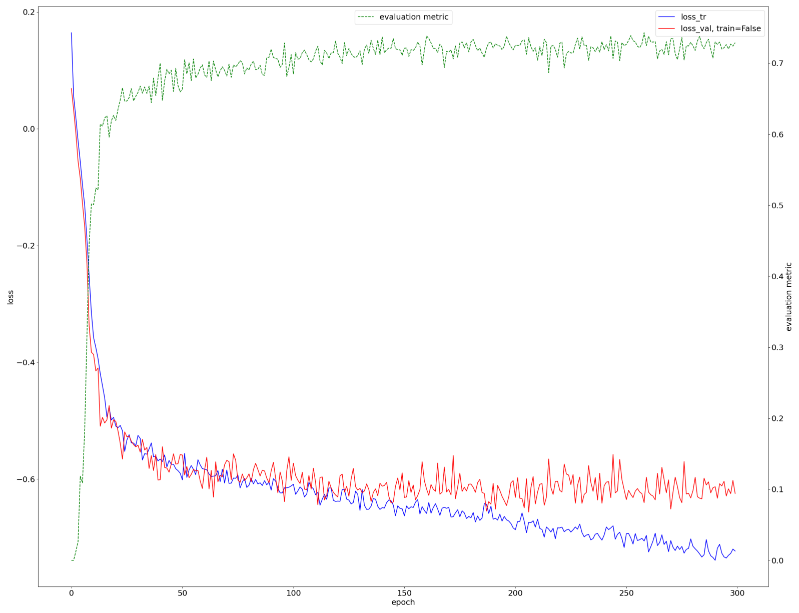

Supplement: Supplementary Data 3 [file mmc3.pdf]
