# Supplementary material for: AID-RT: Standardising Artificial Intelligence Documentation in RadioTherapy with a domain-specific model card
Source: Phys Imaging Radiat Oncol. 2026 Mar 6;38:100940. doi: 10.1016/j.phro.2026.100940 (PMC12997223; doi:10.1016/j.phro.2026.100940)
Supplement: Supplementary Data 4 [file mmc4.pdf]

Model Card — version 0.1 — Model DOSEPRED_PT_ORO_5425700Gy_CTrecons

Task: Dose prediction

---

0. Card Metadata

Creation date: 2025/08/25

Versioning

- **Version number**: 0.1
- **Version changes**: NA

---

1. Model Basic Information

Name: DOSEPRED_PT_ORO_5425700Gy_CTrecons

Creation date: 2024/08/01

Versioning

- **Version number**: 01.00.0000
- **Version changes**: NA

Model scope

- **Summary**: Prediction of 3D dose distribution for proton therapy treatments of oropharyngeal cancer with direct quantification of global uncertainty
- **Anatomical site**: HN

Clearance

- **Type**: Not approved for medical use - only research use

Approved by

- **Name(s)**: NA
- **Institution(s)**: NA
- **Contact email(s)**: NA

Additional Information

NA

Intended users: Radiation oncologists, medical physicists, and dosimetrists

Observed limitations: Model only valid for specific beam configuration of 4 beams

Potential limitations: Might fail for prescriptions different from two level 54.25/70Gy

Type of learning architecture: UNet

Developed by

- **Name**: Margerie Huet Dastarac
- **Institution(s)**: UCLouvain
- **Contact email(s)**: margerie.huet@gmail.com

Conflict of interest: NA

Software licence: Apache 2.0

Code source: https://gitlab.com/ai4miro/ct-reconstruction-for-uncertainty-quatification-of-hdunet

Citation details: Huet-Dastarac M, Nguyen D, Longton E, Jiang S, Lee J, Montero AB. Can input reconstruction be used to directly estimate uncertainty of a dose prediction U-Net model? Med Phys. 2024; 51: 7369–7377. https://doi.org/10.1002/mp.17287

URL info: https://aapm.onlinelibrary.wiley.com/doi/abs/10.1002/mp.17287

---

2. Technical specifications

2.1 Model overview

Model pipeline

- **Summary:** Proton (IMPT) Dose prediction with UNet architecture for bilateral head and neck cancer patients (54.25/70Gy prescription). Inputs to the model are CT, binary masks of the OARS, and target prescription values (all in nifti format nii.gz), and output is a three-dimensional dose distribution (.nii.gz) and reconstructed CT. The reconstructed CT is used as uncertainty proxy when compared to the real, input CT
- **Model inputs:** ['CT', 'RTSTRUCT_CTV', 'RTSTRUCT_Brainstem', 'RTSTRUCT_SpinalCord', 'RTSTRUCT_Parotid_L', 'RTSTRUCT_Parotid_R', 'RTSTRUCT_Esophagus_S', 'RTSTRUCT_Glottis', 'RTSTRUCT_Cavity_Oral', 'RTSTRUCT_Musc_Constrict_I', 'RTSTRUCT_Musc_Constrict_M', 'RTSTRUCT_Musc_Constrict_S', 'RTSTRUCT_Glnd_Submand_L', 'RTSTRUCT_Glnd_Submand_R', 'RTSTRUCT_Larynx_SG']
- **Model outputs:** ['RTDOSE', 'CT']
- **Pre-processing:** "Dental artefact and contrast product removal by overriding with HU=0, remove all dose outside the body contour (set voxel values equal to zero), remove the couch and normalization of CT voxel values. . ",
- **Post-processing:** "Normalization of the target volume dose median to the prescribed dose"

2.2 Learning architecture(s)

Learning architecture 1

Field	Value
**Total number of trainable parameters**	2512080
**Number of inputs**	14
**Input content**	—
**Input format**	[float,float,bool,bool,bool,bool,bool,bool,bool,bool,bool,bool,bool,bool,bool,bool]
**Input size**	[128,128,128]
**Number of outputs**	2
**Output content**	—
**Output format**	[float, float]
**Output size**	[[128,128,128],[128,128,128]]
**Loss function**	MSE
**Batch size**	1
**Regularisation**	Dropout
**Uncertainty quantification techniques**	Addition of a branch stemming from the bottleneck of the UNet architecture. This branch reconstructs the CT scan given as input. The input reconstruction error (MSE) can be used as a surrogate of the model uncertainty.
**Explainability techniques**	NA
**Citation(s)**	Huet-Dastarac M, Nguyen D, Longton E, Jiang S, Lee J, Montero AB. Can input reconstruction be used to directly estimate uncertainty of a dose prediction U-Net model? Med Phys. 2024; 51: 7369–7377. https://doi.org/10.1002/mp.17287

2.3 Hardware & software

- **Libraries and dependencies:** Monai, Pytorch
- **Hardware (recommended):** NVIDIA RTX 3060 or similar mid-range GPU (for inference)
- **Inference time (s) for recommended hardware:** 10

3. Training Data Methodology and Information

Fine tuned form

- **Model name:** NA
- **URL/DOI to model card:** NA
- **Tuning technique:** NA

Training Dataset

General information

- **Total size:** [55,55,55,55,55]
- **Number of patients:** 55
- **Source:** Private dataset from Cliniques Universitaires Saint Luc and UCLouvain
- **Acquisition period:** 2019-2020
- **Inclusion / exclusion criteria:** Inclusion: oropharyngeal patients, bilateral cases Exclusion: anything outside this location and unilateral cases, post-operative patients were also excluded, patients before 2015 were not included because they did not comply with current delineation guidelines for OAR (Brouwer et al.)
- **Type of data augmentation:** [Homotecia by the sagittal plane, patching]
- **Strategy for data augmentation:** Included in the training loop where transformation were performed randomly on the fly

Technical specifications

CT (model_inputs)

Field	Value
**Image resolution**	[3mm,3mm,3mm]
**Patient positioning**	Head First Supine
**Scan(s) manufacturer and model**	NA
**Scan acquisition parameters**	NA
**Scan reconstruction parameters**	NA
**FOV**	NA

RTSTRUCT_CTV (model_inputs)

Field	Value
**Image resolution**	[3mm,3mm,3mm]
**Patient positioning**	Head First Supine

Field	Value
**Scan(s) manufacturer and model**	NA
**Scan acquisition parameters**	NA
**Scan reconstruction parameters**	NA
**FOV**	NA

RTSTRUCT_Brainstem (model_inputs)

Field	Value
**Image resolution**	[3mm,3mm,3mm]
**Patient positioning**	Head First Supine
**Scan(s) manufacturer and model**	—
**Scan acquisition parameters**	—
**Scan reconstruction parameters**	—
**FOV**	—

RTSTRUCT_SpinalCord (model_inputs)

Field	Value
**Image resolution**	[3mm,3mm,3mm]
**Patient positioning**	Head First Supine
**Scan(s) manufacturer and model**	—
**Scan acquisition parameters**	—
**Scan reconstruction parameters**	—
**FOV**	—

RTSTRUCT_Parotid_L (model_inputs)

Field	Value
**Image resolution**	[3mm,3mm,3mm]
**Patient positioning**	Head First Supine
**Scan(s) manufacturer and model**	—
**Scan acquisition parameters**	—
**Scan reconstruction parameters**	—
**FOV**	—

RTSTRUCT_Parotid_R (model_inputs)

Field	Value
**Image resolution**	[3mm,3mm,3mm]
**Patient positioning**	Head First Supine
**Scan(s) manufacturer and model**	—
**Scan acquisition parameters**	—
**Scan reconstruction parameters**	—
**FOV**	—

RTSTRUCT_Esophagus_S (model_inputs)

Field	Value
**Image resolution**	[3mm,3mm,3mm]
**Patient positioning**	Head First Supine
**Scan(s) manufacturer and model**	—
**Scan acquisition parameters**	—
**Scan reconstruction parameters**	—
**FOV**	—

RTSTRUCT_Glottis (model_inputs)

Field	Value
**Image resolution**	[3mm,3mm,3mm]
**Patient positioning**	Head First Supine
**Scan(s) manufacturer and model**	—
**Scan acquisition parameters**	—
**Scan reconstruction parameters**	—
**FOV**	—

RTSTRUCT_Cavity_Oral (model_inputs)

Field	Value
**Image resolution**	[3mm,3mm,3mm]
**Patient positioning**	Head First Supine
**Scan(s) manufacturer and model**	—

Field	Value
**Scan acquisition parameters**	—
**Scan reconstruction parameters**	—
**FOV**	—

RTSTRUCT_Musc_Constrict_I (model_inputs)

Field	Value
**Image resolution**	[3mm,3mm,3mm]
**Patient positioning**	Head First Supine
**Scan(s) manufacturer and model**	—
**Scan acquisition parameters**	—
**Scan reconstruction parameters**	—
**FOV**	—

RTSTRUCT_Musc_Constrict_M (model_inputs)

Field	Value
**Image resolution**	[3mm,3mm,3mm]
**Patient positioning**	Head First Supine
**Scan(s) manufacturer and model**	—
**Scan acquisition parameters**	—
**Scan reconstruction parameters**	—
**FOV**	—

RTSTRUCT_Musc_Constrict_S (model_inputs)

Field	Value
**Image resolution**	[3mm,3mm,3mm]
**Patient positioning**	Head First Supine
**Scan(s) manufacturer and model**	—
**Scan acquisition parameters**	—
**Scan reconstruction parameters**	—
**FOV**	—

RTSTRUCT_Glnd_Submand_L (model_inputs)

Field	Value
**Image resolution**	[3mm,3mm,3mm]
**Patient positioning**	Head First Supine
**Scan(s) manufacturer and model**	—
**Scan acquisition parameters**	—
**Scan reconstruction parameters**	—
**FOV**	—

RTSTRUCT_Glnd_Submand_R (model_inputs)

Field	Value
**Image resolution**	[3mm,3mm,3mm]
**Patient positioning**	Head First Supine
**Scan(s) manufacturer and model**	—
**Scan acquisition parameters**	—
**Scan reconstruction parameters**	—
**FOV**	—

RTSTRUCT_Larynx_SG (model_inputs)

Field	Value
**Image resolution**	[3mm,3mm,3mm]
**Patient positioning**	Head First Supine
**Scan(s) manufacturer and model**	—
**Scan acquisition parameters**	—
**Scan reconstruction parameters**	—
**FOV**	—

RTDOSE (model_outputs)

Field	Value
**Image resolution**	[3mm,3mm,3mm]
**Patient positioning**	Head First Supine
**Scan(s) manufacturer and model**	NA
**Scan acquisition parameters**	NA

Field	Value
**Scan reconstruction parameters**	NA
**FOV**	NA

CT (model_outputs)

Field	Value
**Image resolution**	[3mm,3mm,3mm]
**Patient positioning**	Head First Supine
**Scan(s) manufacturer and model**	NA
**Scan acquisition parameters**	NA
**Scan reconstruction parameters**	NA
**FOV**	NA

- **Treatment modality:** External beam radiation therapy - Protons - Scanning beam multi-field optimization
- **Beam configuration and energy:** four beams at (10, 60), (10, 120), (350, 240), and (350, 300) for couch and gantry angles
- **Dose engine:** Monte Carlo
- **Target volumes and prescription:** [CTV_5425, CTV_7000]
- **Number of fractions:** [35]
- **Reference standard:** In-house planning protocol https://aapm.onlinelibrary.wiley.com/action/downloadSupplement?doi=10.1002%2Fmp.16408&file=mp16408-sup-0001-SuppMat.pdf
- **Reference standard QA:** All plans were consistently planned by the same radiation therapy technician (RTT) following an Internal protocol
- **Additional information:** All treatments are planned in the treatment planning system (TPS) RayStation 11B (Raysearch Laboratories, AB) with a research license. The plans were generated through multiple robust optimization iterations (range error = 2.6%, setup error = 4 mm) with 21 (3x7) uncertainty scenarios and a final Monte Carlo (MC) dose calculation with an uncertainty of 1%. The dose grid was 3x3x3 mm3.

Patient demographics and clinical characteristics

- **ICD10/11:** C10.9
- **TNM staging:** NA
- **Age:** NA
- **Sex:** NA

Validation strategy: 5-fold cross validation

Validation data partition: 15%

Weights initialization: Uniform **Epochs:** [250] **Optimiser:** Adam **Learning rate:** 1e-4

Model choice criteria: Last epoch

Inference method: Sliding window over patches

4. Evaluation Data Methodology, Results and Commissioning

1 Evaluation from original paper

Evaluation date: 2024/06/01

Evaluated by

- **Name(s):** NA
- **Institution(s):** NA
- **Contact email(s):** NA
- **Same as 'Approved by':** Yes

Evaluation frame: Retrospective

Evaluation dataset

General information

- **Total size:** 55
- **Number of patients:** 55
- **Source:** Private dataset from Cliniques Universitaires Saint Luc and UCLouvain
- **Acquisition period:** 2019-2020
- **Inclusion / Exclusion criteria:** Inclusion: oropharyngeal patients, bilateral cases Exclusion: anything outside this location and unilateral cases, post-operative patients were also excluded, patients before 2015 were not included because they did not comply with current delineation guidelines for OAR (Brouwer et al.)
- **URL info:** —

Technical specifications

CT (model_inputs)

Field	Value
**Image resolution**	[3mm,3mm,3mm]
**Patient positioning**	Head First Supine
**Scan(s) manufacturer and model**	—
**Scan acquisition parameters**	—
**Scan reconstruction parameters**	—
**FOV**	—

RTSTRUCT_CTV (model_inputs)

Field	Value
**Image resolution**	[3mm,3mm,3mm]
**Patient positioning**	Head First Supine
**Scan(s) manufacturer and model**	—
**Scan acquisition parameters**	—
**Scan reconstruction parameters**	—
**FOV**	—

RTSTRUCT_Brainstem (model_inputs)

Field	Value
**Image resolution**	[3mm,3mm,3mm]
**Patient positioning**	Head First Supine
**Scan(s) manufacturer and model**	—
**Scan acquisition parameters**	—
**Scan reconstruction parameters**	—
**FOV**	—

RTSTRUCT_SpinalCord (model_inputs)

Field	Value
**Image resolution**	[3mm,3mm,3mm]
**Patient positioning**	Head First Supine
**Scan(s) manufacturer and model**	—
**Scan acquisition parameters**	—
**Scan reconstruction parameters**	—
**FOV**	—

RTSTRUCT_Parotid_L (model_inputs)

Field	Value
**Image resolution**	[3mm,3mm,3mm]
**Patient positioning**	Head First Supine
**Scan(s) manufacturer and model**	—
**Scan acquisition parameters**	—

Field	Value
**Scan reconstruction parameters**	—
**FOV**	—

RTSTRUCT_Parotid_R (model_inputs)

Field	Value
**Image resolution**	[3mm,3mm,3mm]
**Patient positioning**	Head First Supine
**Scan(s) manufacturer and model**	—
**Scan acquisition parameters**	—
**Scan reconstruction parameters**	—
**FOV**	—

RTSTRUCT_Esophagus_S (model_inputs)

Field	Value
**Image resolution**	[3mm,3mm,3mm]
**Patient positioning**	Head First Supine
**Scan(s) manufacturer and model**	—
**Scan acquisition parameters**	—
**Scan reconstruction parameters**	—
**FOV**	—

RTSTRUCT_Glottis (model_inputs)

Field	Value
**Image resolution**	[3mm,3mm,3mm]
**Patient positioning**	Head First Supine
**Scan(s) manufacturer and model**	—
**Scan acquisition parameters**	—
**Scan reconstruction parameters**	—
**FOV**	—

RTSTRUCT_Cavity_Oral (model_inputs)

Field	Value
**Image resolution**	[3mm,3mm,3mm]
**Patient positioning**	Head First Supine
**Scan(s) manufacturer and model**	—
**Scan acquisition parameters**	—
**Scan reconstruction parameters**	—
**FOV**	—

RTSTRUCT_Musc_Constrict_I (model_inputs)

Field	Value
**Image resolution**	[3mm,3mm,3mm]
**Patient positioning**	Head First Supine
**Scan(s) manufacturer and model**	—
**Scan acquisition parameters**	—
**Scan reconstruction parameters**	—
**FOV**	—

RTSTRUCT_Musc_Constrict_M (model_inputs)

Field	Value
**Image resolution**	[3mm,3mm,3mm]
**Patient positioning**	Head First Supine
**Scan(s) manufacturer and model**	—
**Scan acquisition parameters**	—
**Scan reconstruction parameters**	—
**FOV**	—

RTSTRUCT_Musc_Constrict_S (model_inputs)

Field	Value
**Image resolution**	[3mm,3mm,3mm]
**Patient positioning**	Head First Supine
**Scan(s) manufacturer and model**	—
**Scan acquisition parameters**	—

Field	Value
**Scan reconstruction parameters**	—
**FOV**	—

RTSTRUCT_Glnd_Submand_L (model_inputs)

Field	Value
**Image resolution**	[3mm,3mm,3mm]
**Patient positioning**	Head First Supine
**Scan(s) manufacturer and model**	—
**Scan acquisition parameters**	—
**Scan reconstruction parameters**	—
**FOV**	—

RTSTRUCT_Glnd_Submand_R (model_inputs)

Field	Value
**Image resolution**	[3mm,3mm,3mm]
**Patient positioning**	Head First Supine
**Scan(s) manufacturer and model**	—
**Scan acquisition parameters**	—
**Scan reconstruction parameters**	—
**FOV**	—

RTSTRUCT_Larynx_SG (model_inputs)

Field	Value
**Image resolution**	[3mm,3mm,3mm]
**Patient positioning**	Head First Supine
**Scan(s) manufacturer and model**	—
**Scan acquisition parameters**	—
**Scan reconstruction parameters**	—
**FOV**	—

RTDOSE (model_outputs)

Field	Value
**Image resolution**	[3mm,3mm,3mm]
**Patient positioning**	Head First Supine
**Scan(s) manufacturer and model**	NA
**Scan acquisition parameters**	NA
**Scan reconstruction parameters**	NA
**FOV**	NA

CT (model_outputs)

Field	Value
**Image resolution**	[3mm,3mm,3mm]
**Patient positioning**	Head First Supine
**Scan(s) manufacturer and model**	NA
**Scan acquisition parameters**	NA
**Scan reconstruction parameters**	NA
**FOV**	NA

- **Dose engine:** Monte Carlo
- **Target volumes and prescription:** [CTV_5425, CTV_7000]
- **Number of fractions:** 35
- **Reference standard:** In-house planning protocol https://aapm.onlinelibrary.wiley.com/action/downloadSupplement?doi=10.1002%2Fmp.16408&file=mp16408-sup-0001-SuppMat.pdf
- **Reference standard QA:** All plans were consistently planned by the same radiation therapy technician (RTT) following an Internal protocol
- **Additional information:** All treatments are planned in the treatment planning system (TPS) RayStation 11B (Raysearch Laboratories, AB) with a research license. The plans were generated through multiple robust optimization iterations (range error = 2.6%, setup error = 4 mm) with 21 (3x7) uncertainty scenarios and a final Monte Carlo (MC) dose calculation with an uncertainty of 1%. The dose grid was 3x3x3 mm3.

Patient demographics and clinical characteristics

- **ICD10/11:** C10.9
- **Age:** —
- **Sex:** —
- **Additional information:** See appendix Fig2

Quantitative evaluation

Dose Metrics

D99 error CTV7000

Field	Value
Type	D99 error CTV7000
Metric Specifications	NA
On Volume	CTV
Dose Grid Resolution	[3mm,3mm,3mm]
Sample Data	—
Mean Data	[-0.143, -5.248, 2.124, 1.260]
Figure DM DP Appendix Label	Fig2

D95 error CTV7000

Field	Value
Type	D95 error CTV7000
Metric Specifications	NA
On Volume	CTV
Dose Grid Resolution	[3mm,3mm,3mm]
Sample Data	—
Mean Data	[-0.452, -12.129, 2.395, 2.237]
Figure DM DP Appendix Label	—

D95 error CTV5425

Field	Value
Type	D95 error CTV5425
Metric Specifications	NA
On Volume	CTV
Dose Grid Resolution	[3mm,3mm,3mm]
Sample Data	—
Mean Data	[0.108, -5.104, 1.846, 0.975]

Field	Value
Figure DM DP Appendix Label	—

D99 error CTV5425

Field	Value
Type	D99 error CTV5425
Metric Specifications	NA
On Volume	CTV
Dose Grid Resolution	[3mm,3mm,3mm]
Sample Data	—
Mean Data	[0.199, -1.387, 1.639, 0.661]
Figure DM DP Appendix Label	—

D2 error Brainstem

Field	Value
Type	D2 error Brainstem
Metric Specifications	NA
On Volume	Brainstem
Dose Grid Resolution	[3mm,3mm,3mm]
Sample Data	—
Mean Data	[0.015, -4.374, 3.448, 1.448]
Figure DM DP Appendix Label	—

Dmean error Esophagus_S

Field	Value
Type	Dmean error Esophagus_S
Metric Specifications	NA
On Volume	Esophagus_S
Dose Grid Resolution	[3mm,3mm,3mm]
Sample Data	—
Mean Data	[-0.035, -5.757, 11.057, 2.046]
Figure DM DP Appendix Label	—

Dmean error Cavity_Oral

Field	Value
Type	Dmean error Cavity_Oral
Metric Specifications	NA
On Volume	Cavity_Oral
Dose Grid Resolution	[3mm,3mm,3mm]
Sample Data	—
Mean Data	[-0.072, -2.943, 2.286, 1.053]
Figure DM DP Appendix Label	—

Dmean error Musc_Constrict_I

Field	Value
Type	Dmean error Musc_Constrict_I
Metric Specifications	NA
On Volume	Musc_Constrict_I
Dose Grid Resolution	[3mm,3mm,3mm]
Sample Data	—
Mean Data	[-0.069, -4.462, 3.279, 1.300]
Figure DM DP Appendix Label	—

Dmean error Musc_Constrict_M

Field	Value
Type	Dmean error Musc_Constrict_M
Metric Specifications	NA
On Volume	Musc_Constrict_M
Dose Grid Resolution	[3mm,3mm,3mm]
Sample Data	—
Mean Data	[-0.027, -3.836, 4.195, 1.269]
Figure DM DP Appendix Label	—

Dmean error Musc_Constrict_S

Field	Value
Type	Dmean error Musc_Constrict_S
Metric Specifications	NA
On Volume	Musc_Constrict_S
Dose Grid Resolution	[3mm,3mm,3mm]
Sample Data	—
Mean Data	[0.005, -2.293, 2.968, 1.146]
Figure DM DP Appendix Label	—

Dmean error Parotid_L

Field	Value
Type	Dmean error Parotid_L
Metric Specifications	NA
On Volume	Parotid_L
Dose Grid Resolution	[3mm,3mm,3mm]
Sample Data	—
Mean Data	[-0.052, -2.274, 2.975, 1.122]
Figure DM DP Appendix Label	—

Dmean error Parotid_R

Field	Value
Type	Dmean error Parotid_R
Metric Specifications	NA
On Volume	Parotid_R
Dose Grid Resolution	[3mm,3mm,3mm]
Sample Data	—
Mean Data	[-0.048, -2.258, 2.053, 0.933]
Figure DM DP Appendix Label	—

Dmean error Glnd_Submand_L

Field	Value
Type	Dmean error Glnd_Submand_L

Field	Value
Metric Specifications	NA
On Volume	Glnd_Submand_L
Dose Grid Resolution	[3mm,3mm,3mm]
Sample Data	—
Mean Data	[0.007, -3.826, 4.169, 1.647]
Figure DM DP Appendix Label	—

Dmean error Glnd_Submand_R

Field	Value
Type	Dmean error Glnd_Submand_R
Metric Specifications	NA
On Volume	Glnd_Submand_R
Dose Grid Resolution	[3mm,3mm,3mm]
Sample Data	—
Mean Data	[0.131, -3.455, 4.942, 1.854]
Figure DM DP Appendix Label	—

D2 error SpinalCord

Field	Value
Type	D2 error SpinalCord
Metric Specifications	NA
On Volume	SpinalCord
Dose Grid Resolution	[3mm,3mm,3mm]
Sample Data	—
Mean Data	[-0.079, -5.246, 3.804, 1.840]
Figure DM DP Appendix Label	—

D5 error Larynx_SG

Field	Value
Type	D5 error Larynx_SG
Metric Specifications	NA

Field	Value
On Volume	Larynx_SG
Dose Grid Resolution	[3mm,3mm,3mm]
Sample Data	—
Mean Data	[-0.273, -6.216, 3.893, 2.196]
Figure DM DP Appendix Label	—

Qualitative evaluation

Evaluators information: —

Likert scoring

- Method: —
- Results: —

Turing test

- Method: —
- Results: —

Time saving

- Method: —
- Results: —

Other

- Method: —
- Results: —

Explainability: —

Citation details: —

---

5. Other considerations

No other considerations provided.

Appendix

appendix_84fe8c14_Fig1.jpg

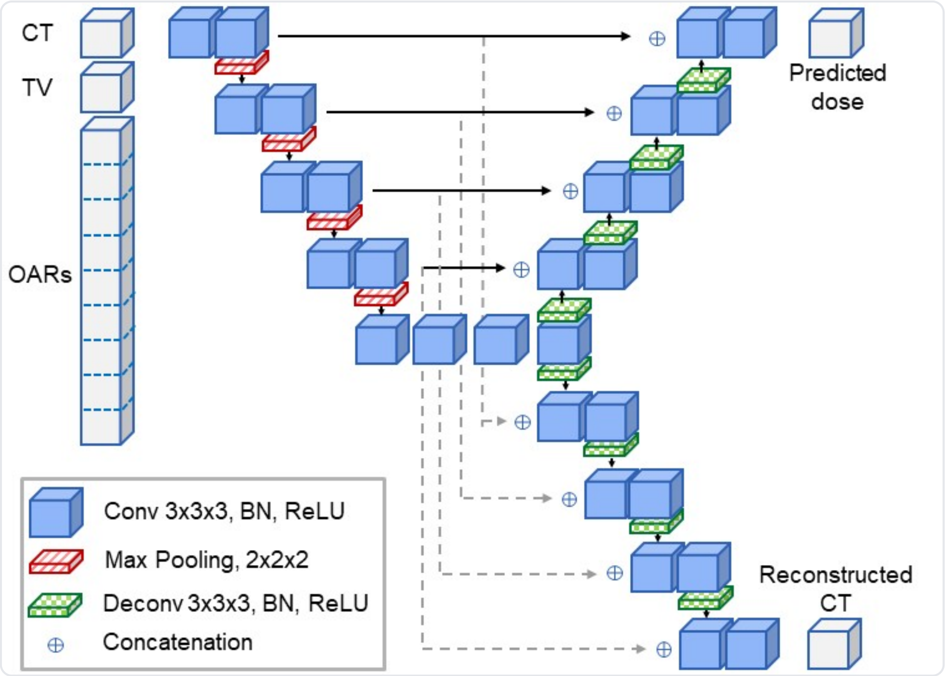

appendix_de9c9b70_Fig2.jpg

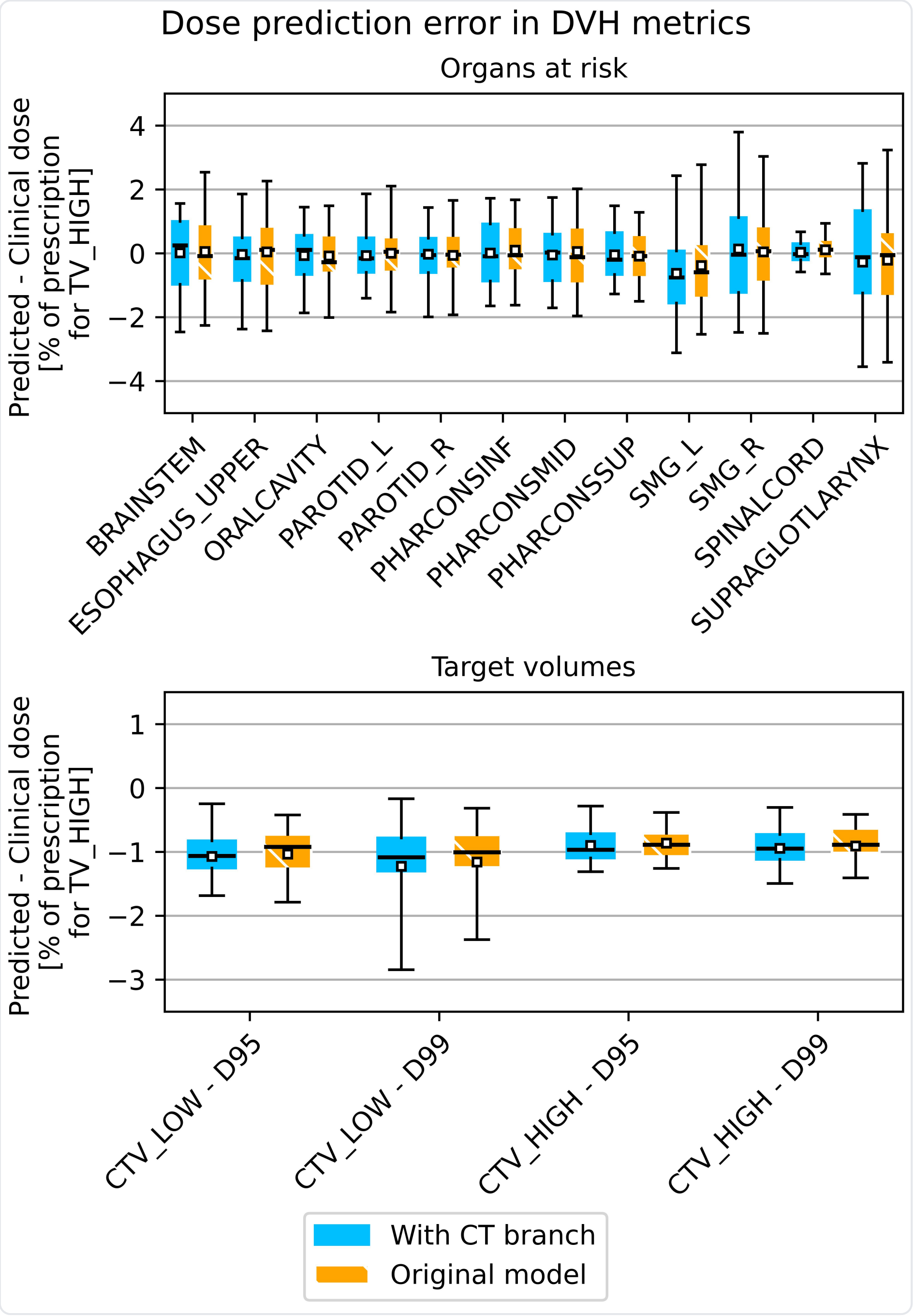

Dose prediction error in DVH metrics
Organs at risk
Predicted - Clinical dose [% of prescription for TV_HIGH]
4
2
0
−2
−4
BRAINSTEM
ESOPHAGUS_UPPER
ORALCAVITY
PAROTID_L
PAROTID_R
PHARCONSINF
PHARCONSMID
PHARCONSSUP
SMG_L
SMG_R
SPINALCORD
SUPRAGLOTLARYNX
Target volumes
Predicted - Clinical dose [% of prescription for TV_HIGH]
1
0
−1
−2
−3
CTV_LOW - D95
CTV_LOW - D99
CTV_HIGH - D95
CTV_HIGH - D99
With CT branch
Original model
